# De Novo Design of Spiro-Type Hole-Transporting Material: Anisotropic Regulation Toward Efficient and Stable Perovskite Solar Cells

**DOI:** 10.34133/research.0332

**Published:** 2024-03-23

**Authors:** Xuran Wang, Mingliang Wang, Zilong Zhang, Dong Wei, Shidong Cai, Yuheng Li, Rui Zhang, Liangliang Zhang, Ruidan Zhang, Chenhui Zhu, Xiaozhen Huang, Feng Gao, Peng Gao, Yang Wang, Wei Huang

**Affiliations:** ^1^ Strait Institute of Flexible Electronics (SIFE, Future Technologies), Fujian Key Laboratory of Flexible Electronics, Fujian Normal University and Strait Laboratory of Flexible Electronics (SLoFE), Fuzhou, Fujian 350117, China.; ^2^ College of Physics and Energy, Fujian Normal University, Fuzhou, Fujian 350117, China.; ^3^ CAS Key Laboratory of Design and Assembly of Functional Nanostructures, and Fujian Provincial Key Laboratory of Nanomaterials Fujian Institute of Research on the Structure of Matter, Chinese Academy of Sciences, Fuzhou, Fujian 350002, China.; ^4^ Xiamen Key Laboratory of Rare Earth Photoelectric Functional Materials, Xiamen Institute of Rare Earth Materials, Haixi Institute, Chinese Academy of Sciences, Xiamen 361021, China.; ^5^ Department of Physics, Chemistry and Biology (IFM), Linköping University, Linköping, Sweden.; ^6^ Advanced Light Source, Lawrence Berkeley National Laboratory, Berkeley, CA 94720, USA.; ^7^ Frontiers Science Center for Flexible Electronics (FSCFE), MIIT Key Laboratory of Flexible Electronics (KLoFE), Northwestern Polytechnical University, Xi’an710072, China.; ^8^ Key Laboratory of Flexible Electronics (KLOFE) and Institute of Advanced Materials (IAM), Nanjing Tech University (NanjingTech), Nanjing211800, China.

## Abstract

2,2′,7,7′-Tetrakis(*N*,*N*-di-p-methoxyphenyl)-amine-9,9′-spirobifluorene (Spiro-OMeTAD) represents the state-of-the-art hole-transporting material (HTM) in n-i-p perovskite solar cells (PSCs). However, its susceptibility to stability issues has been a long-standing concern. In this study, we embark on a comprehensive exploration of the untapped potential within the family of spiro-type HTMs using an innovative anisotropic regulation strategy. Diverging from conventional approaches that can only modify spirobifluorene with single functional group, this approach allows us to independently tailor the two orthogonal components of the spiro-skeleton at the molecular level. The newly designed HTM, SF-MPA-MCz, features enhanced thermal stability, precise energy level alignment, superior film morphology, and optimized interfacial properties when compared to Spiro-OMeTAD, which contribute to a remarkable power conversion efficiency (PCE) of 24.53% for PSCs employing SF-MPA-MCz with substantially improved thermal stability and operational stability. Note that the optimal concentration for SF-MPA-MCz solution is only 30 mg/ml, significantly lower than Spiro-OMeTAD (>70 mg/ml), which could remarkably reduce the cost especially for large-area processing in future commercialization. This work presents a promising avenue for the versatile design of multifunctional HTMs, offering a blueprint for achieving efficient and stable PSCs.

## Introduction

Organic–inorganic halide perovskite solar cells (PSCs) have attracted well-deserved attention since their introduction in 2009, which exhibited a significant increase in their power conversion efficiencies (PCEs) from 3.8% to over 26% nowadays [1,2]. To expedite their commercialization, the stability issues originating from the soft structure of perovskite (PVK) materials, the unideal interfacial contact, and the unstable nature of other functional layers have to be addressed. Generally, the top-performing PSCs feature a n-i-p structure with metal oxides as an electron-transporting layer underneath the perovskite and organic p-type semiconductors as a hole-transporting layer (HTL) above the perovskite. Up to date, 2,2′,7,7′-tetrakis(*N*,*N*-di-p-methoxyphenyl)-amine-9,9′-spirobifluorene (Spiro-OMeTAD) has proven to be the most efficient and universal hole-transporting material (HTM) utilized in n-i-p PSCs mainly due to its relatively straightforward synthesis and amorphous film morphology [3]. However, high dopant concentration involving hygroscopic lithium salts or volatile additives is unavoidably needed to improve the hole conductivity of Spiro-OMeTAD film, which will undoubtedly sacrifice the device stability [4,5]. Hence, exploring appropriate HTMs to enhance device stability while maintaining the high efficiency of PSCs is still in urgent need at the present stage.

**Fig. 1.**
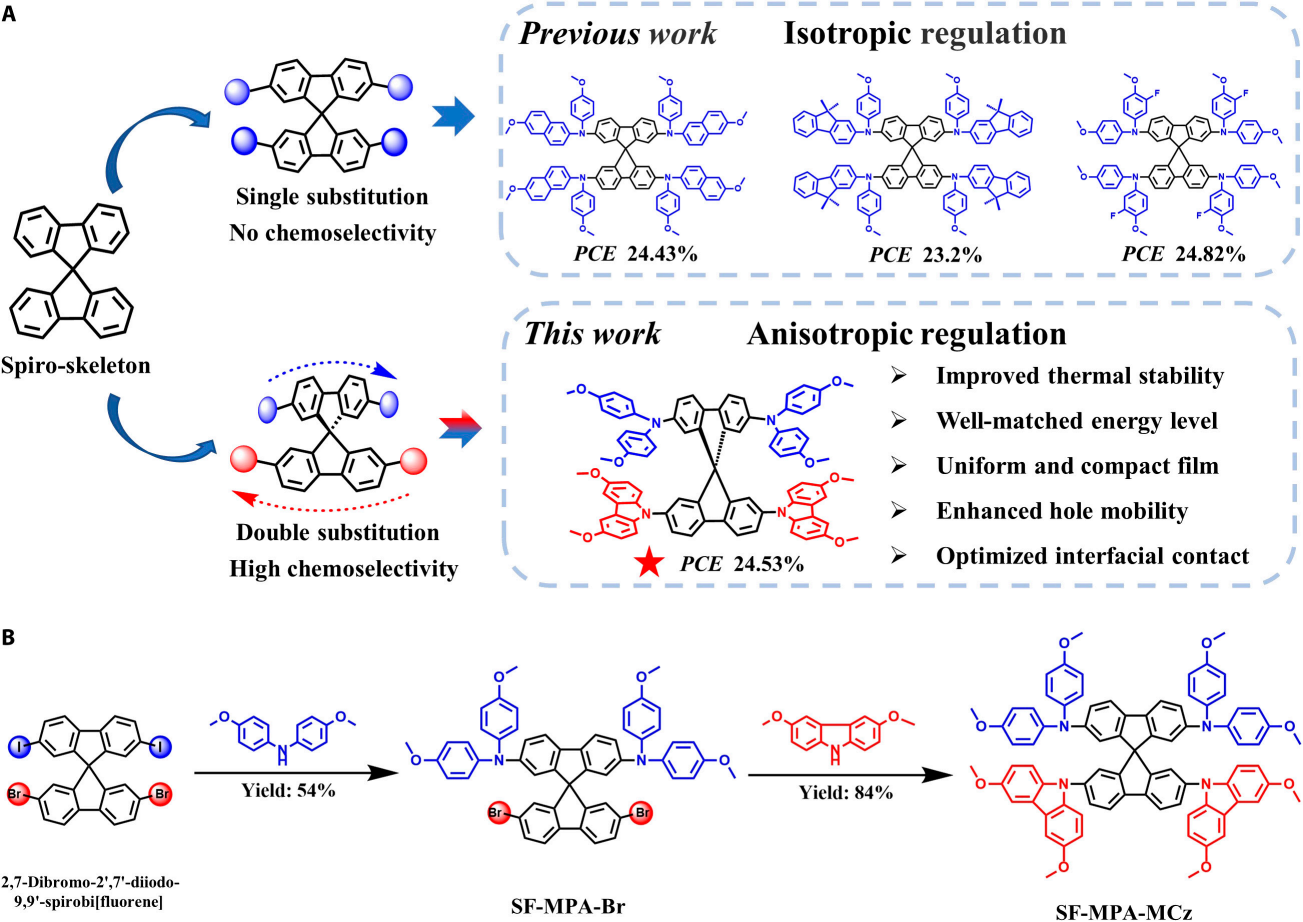
Molecular design concept and synthesis route. (A) Isotropic and anisotropic regulation for the spirobifluorene-based molecules. (B) Synthetic route for SF-MPA-MCz.

In retrospect, to remove the use of dopants, numerous efforts have been devoted to exploiting new HTMs with high intrinsic hole mobility and good passivation effect, which indeed show enhanced stability in PSCs [6,7]. Nevertheless, the device performance incorporating these dopant-free HTMs is still far behind the best-in-class devices based on Spiro-OMeTAD [8–14]. This conversely triggers further research on molecular design and optimization of such spiro-type HTMs to tune their energy levels, structural stability, molecular packing, hole conductivity, and film morphological properties. One approach is to alter the kernel structure of Spiro-OMeTAD (i.e.*,* 9,9′-spirobi[fluorene]) by inserting heteroatoms or reconstructing spiral rings [15–19]. The other approach is to regulate the peripheral fragments of Spiro-OMeTAD [i.e.*,* bis(4-methoxyphenyl)amine] by adopting various strategies such as conjugation extension [20,21], fluorination, and side-chain engineering [22,23]. Despite the progress on the reconstruction of spiro-type HTMs, there still remain several drawbacks: (a) it requires a high concentration of HTM solutions to ensure dense and pin-hole-free films, which increases the consumption of materials and the related preparation cost; (b) high doping concentration can still not be avoided to achieve the maximum efficiency, which may ultimately affect the long-term stability of devices. To clarify this problem, we speculate that the flexibility of molecular design may determine the freedom of regulation on the corresponding properties of HTMs. Specifically, it was found that most work is limited by isotropic regulation methods toward spiro-type molecules. That is, four sites of spirobifluorene are functionalized by the same fragment without changing the molecular symmetry [20,22,24] (Fig. 1A). In this way, although the electronic and film morphological properties of HTMs could be tuned by designing different peripheral groups, the functional diversity of spiro-skeleton is constricted due to single substitution channel, which may pose certain limitation to the selectivity and flexibility of molecular design and structure–property modulation.

Here, we propose an anisotropic regulation strategy based on the high chemoselectivity of the spiro-skeleton due to the different reactivities of halogens during C–N coupling reactions (Fig. 1B). This reactivity discrepancy enables us to independently modify the two orthogonal parts of the spiro-skeleton, providing more opportunities for multifunctional design and flexible regulation of HTMs. As a proof of concept, a novel HTM, namely, SF-MPA-MCz, was developed by tailoring spirobifluorene simultaneously with diphenylamine and carbazole moieties. Unlike the previous work involving isotropic regulation using the single functional unit, we first realized the synchronous regulation based on the spiro-skeleton by incorporating different functional fragments at the single molecular level. Benefiting from this strategy, we found that tiny variation induced by the half replacement of diphenylamine units in Spiro-OMeTAD by carbazole units can result in distinct optoelectronic properties and film morphologies. Encouragingly, as expected, by the introduction of rigid carbazole units, SF-MPA-MCz exhibits improved thermal stability and hole mobility, suitable energy level alignment, excellent film morphology, and optimized interfacial contact, all of which contribute to remarkably high PCE of 24.53% [open voltage (*V*_OC_) of 1.18 V, short-circuit current density (*J*_SC_) of 26.24 mA cm^−2^, fill factor (*FF*) of 79.22%], outperforming the control device based on Spiro-OMeTAD (22.95%). Importantly, compared to Spiro-OMeTAD, the stability of SF-MPA-MCz-based device can be apparently enhanced with more than 90% PCE maintained after 500 h of continuous illumination at 100 (mW cm^−2^) and more than 85% PCE maintained after 1,000-h heating at 85 °C.

## Results

### Design, synthesis, and structures

To determine the effectiveness of an anisotropic regulation strategy, ab initio molecular design should be simple and comparable. Thus, based on the prototypical spiro-type HTM Spiro-OMeTAD, we selectively changed two diphenylamine units to carbazole units due to their similar molecular size and chemical compositions. The remaining diphenylamine units can maintain the molecular oxidizability and superior hole-transporting ability. At the same time, the introduction of rigid and planar carbazole moieties is anticipated to enhance the thermal stability and intermolecular stacking of HTMs, aiming to optimize the intrinsic hole mobility and film morphology of HTMs. Similar to Spiro-OMeTAD, SF-MPA-MCz can be straightforward to synthesize by using the key intermediate 2,7-dibromo-2′,7′-diiodo-9,9′-spirobi[fluorene], whose synthetic details could be found in the Supplementary Materials (Experimental Section). Relying on the different reactivities of bromine and iodine, bis(4-methoxyphenyl)amine was first selectively coupled with iodinated fluorene under relatively mild reaction conditions to obtain the intermediate SF-MPA-Br in a moderate yield of 54%. Successively, a similar Buchwald C–N coupling reaction was carried out between 3,6-dimethoxy-9*H*-carbazole and SF-MPA-Br to produce the target molecule SF-MPA-MCz in a high yield of 84%. The chemical structure of SF-MPA-MCz was well characterized by ^1^H nuclear magnetic resonance (NMR), ^13^C NMR, matrix-assisted laser desorption ionization time-of-flight (MALDI-TOF) mass spectrometry, and elemental analysis (EA).

**Fig. 2.**
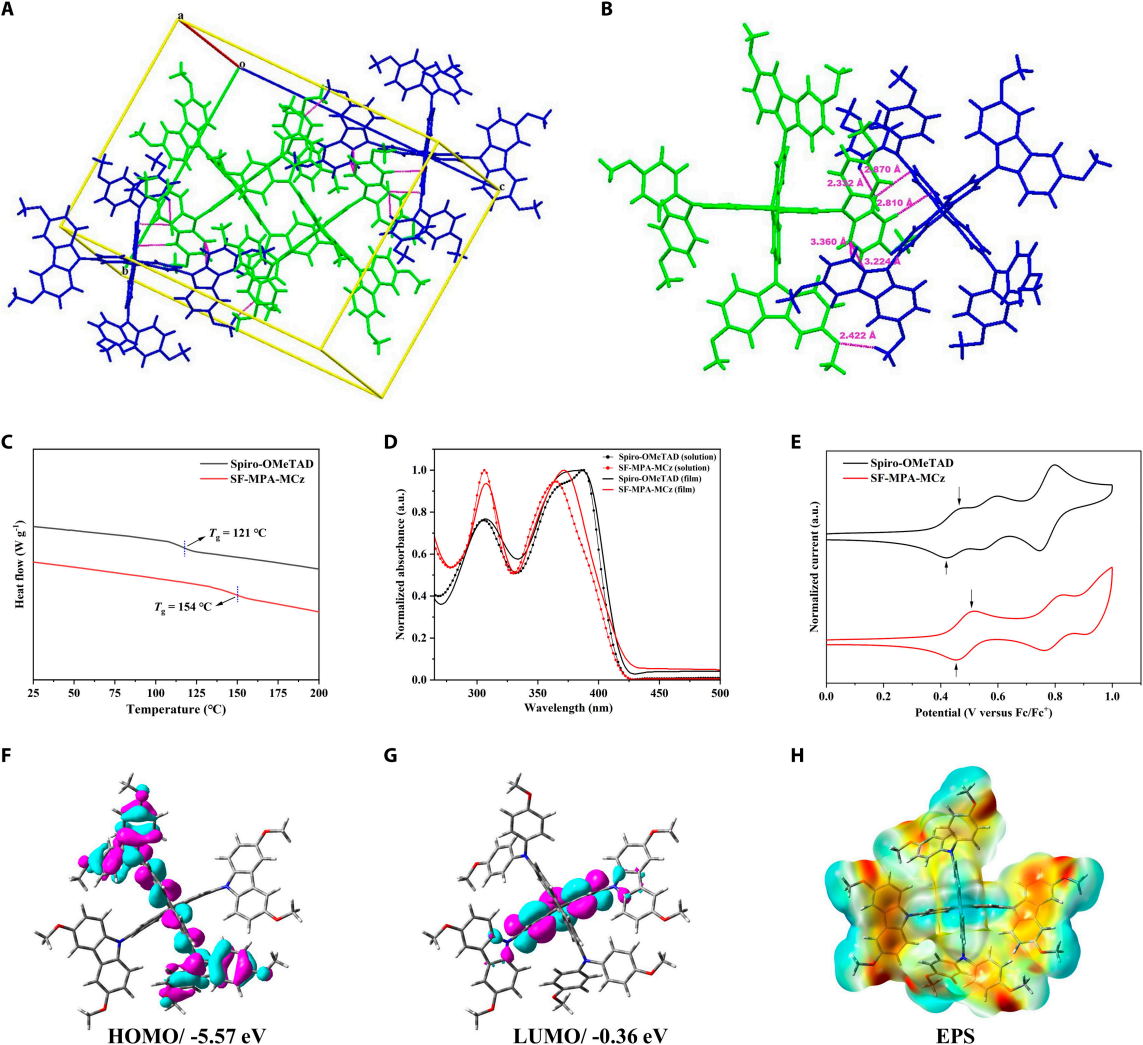
Molecular single-crystal structure and basic optoelectronic properties. (A) Unit cell of the single-crystal structure of SF-MPA-MCz. (B) Dimeric structures within unit cells. (C) DSC curves of Spiro-OMeTAD and SF-MPA-MCz. The vertical dashed lines indicate the glass transition temperature. (D) Normalized UV–vis absorption spectra of Spiro-OMeTAD and SF-MPA-MCz in solution and solid state. (E) Cyclic voltammograms of Spiro-OMeTAD and SF-MPA-MCz. The arrows indicate the first peak cathodic potentials. Illustration of DFT calculated (F) HOMO, (G) LUMO, and (H) EPS mapping for SF-MPA-MCz.

Moreover, the crystal of SF-MPA-MCz can be easily obtained by solvent diffusion method and characterized by single-crystal x-ray diffraction (XRD) to reveal the molecular conformation and stacking (Fig. 2A and B). Spiro-OMeTAD and SF-MPA-MCz belong to the triclinic space group but feature distinct stacking behaviors (Fig. S5 and Table S1). It can be seen that Spiro-OMeTAD contains two molecules in a single-crystal unit cell, while SF-MPA-MCz contains four molecules. As we know, the adjacent Spiro-OMeTAD molecules in a single-crystal unit cell can form parallel π-π stacking by accumulating fluorene structures. However, the steric hindrance effect produced by the peripheral groups and the orthogonal spiro-fluorene will hinder the formation of continuous π-π stacking between each unit cell, which will restrain the delocalization of hole carriers, thereby suppressing carrier transport capacity [25]. By contrast, for SF-MPA-MCz, the terminal bis(4-methoxyphenyl)amine group could be embedded into the adjacent spiro-fluorene cores and, therefore, generate multiple short contacts with adjacent molecules, which may provide continuous channels for intermolecular hole hopping (Fig. S5).

### Thermal, optoelectronic, and thin-film properties

Thermogravimetry analysis (TGA) and differential scanning calorimetry (DSC) were first applied to characterize the thermal properties of HTMs, as shown in (Fig. S6 and Fig. 2C). Expectedly, the incorporation of rigid carbazole units is beneficial for the enhancement of thermal stability of SF-MPA-MCz, which shows a higher decomposition temperature of 445 °C (*T*_d_, 5% weight loss temperature) (Fig. S6) and glass transition temperature (*T*_g_) of 154 °C as opposed to Spiro-OMeTAD (*T*_*d*_* = 437 °C*, *T*_*g*_* = 121 °C*) (Table 1). Figure 2D shows the UV absorption spectra of Spiro-OMeTAD and SF-MPA-MCz. Both HTMs exhibit obvious double absorption peaks in the range of 300 to 400 nm in solutions, but for SF-MPA-MCz, a distinct bathochromic shift is observed from solution to film state, indicating that SF-MPA-MCz has a stronger aggregation than Spiro-OMeTAD in the solid state. Moreover, compared to Spiro-OMeTAD, SF-MPA-MCz exhibits a defined absorption peak ascribed to 0–0 transition at 350 to 400 nm without 0–1 vibrational transition, indicating the different molecular conformation or arrangement for both HTMs [20]. Cyclic voltammetry (CV) was used to evaluate the highest occupied molecular orbital (HOMO) levels of the HTMs in solutions. It can be seen in Fig. 2E that one of the oxidation potential peaks is missing for SF-MPA-MCz due to the reduced number of diphenylamine groups, and the oxidation onset is shifted to a higher potential for SF-MPA-MCz, resulting in deeper HOMO level of −5.17 eV than that of Spiro-OMeTAD (−5.13 eV). To further confirm the energy levels in the film state, ultraviolet photoelectron spectroscopy (UPS) was performed, as shown in Fig. S7. By analyzing the cutoff (*E*_cutoff_) and onset (*E*_onset_) energy regions in the spectra, the HOMO levels of SF-MPA-MCz and Spiro-OMeTAD are calculated to be −5.26 and −5.19 eV, respectively, showing the consistent variation trend with the CV measurements. The lowered HOMO level of SF-MPA-MCz enables better energy level alignment with the perovskite layer, and the enhanced hole transfer may be anticipated, which is favorable for the increment of *V*_OC_ in PSCs.

**Table 1.** Optical, electrochemical, and thermal properties of Spiro-OMeTAD and SF-MPA-MCz

Compounds	*λ*_max_ (nm)		*λ*_onset_ (nm)	*E*_g_^opt^(eV)	*E*_HOMO_^CV^ (eV)	*E*_HOMO_^UPS^ (eV)	*E*_LUMO_^opt^ (eV)	*T*_g_(°C)	*T*_d95_ (°C)	*r*
	Solution	Film	Film							
Spiro-OMeTAD	388	387	422	2.94	−5.13	−5.19	−2.25	121	437^ref^	0.016
SF-MPA-MCz	364	374	430	2.88	−5.17	−5.26	−2.38	154	445	0.080

$E_{g}^{\mathrm{opt}}$* = 1,240/λ*_* onset*_ (film state). *E*_*HOMO*_^*CV*^* = −(E*^*ox*^* – E (Fc/Fc*^*+*^*) + 4.8) eV*. The redox *Fc/Fc*^*+*^ is located at 1.21 V relative to *Ag/Ag*^*+*^. *E*_*HOMO*_^*UPS*^* = Ev = hν − (E*_*cutoff*_* – E*_*onset*_*)*, where the value of *hν* is 21.22 eV. *E*_*LUMO*_^*opt*^* = (E*_*HOMO*_^*UPS*^* + E*_*g*_^*opt*^*)*.

To verify the effectiveness of the anisotropic regulation strategy, fluorescence anisotropy is performed, as shown in Fig. S8 and Table 1. The anisotropy (*r*) value is obtained by the following equation: $r=\frac{I_{\mathrm{VV}}-G\times I_{\mathrm{VH}}}{I_{\mathrm{VV}}+2G\times I_{\mathrm{VH}}}$; here, *V* and *H* represent vertical polarization and horizontal polarization, respectively, and the grating factor (*G*) value is calibrated by the following equation: $G=\frac{I_{\mathrm{HV}}}{I_{\mathrm{HH}}}$ [26]. The obtained *r* value of SF-MPA-MCz is 0.08, much higher than Spiro-OMeTAD with a highly symmetrical structure (0.016), indicating the higher excited state dipole moment and stronger anisotropy of SF-MPA-MCz [27].

To investigate the thin-film properties of two HTMs, atomic force microscopy (AFM) was first carried out. The AFM images of Spiro-OMeTAD and SF-MPA-MCz on the fluorine-doped tin oxide (FTO) exhibit low root-mean-square roughness (*R*_RMS_) values, which means that both HTMs can form smooth film surfaces (Fig. S9A and B). Meanwhile, the film morphology spin-coating on the perovskite is investigated (Fig. S9C and D). Although both HTMs show similar *R*_RMS_, the pin-holes distributed on Spiro-OMeTAD can be clearly observed, which may mitigate the hole transport ability, while SF-MPA-MCz can still exhibit pin-hole free film. Further, the molecular ordering of both HTMs in bulk thin films was studied by two-dimensional (2D) grazing incidence wide-angle x-ray scattering (2D-GIWAXS) (Fig. S10). As can be seen from the diffraction patterns, both HTMs exhibit strong circular diffraction signals without any preferential orientation, indicating the amorphous nature of their thin films. However, it is worth noting that after the annealing process, the π-π stacking distance of SF-MPA-MCz is reduced from 4.90 Å to 4.85 Å, while nearly no change is observed for Spiro-OMeTAD, which suggests that SF-MPA-MCz tends to arrange more ordered molecular stacking in film state and is likely to form dense and stable films (Table S2). The contact angle measurements reveal that SF-MPA-MCz has a higher water contact angle of 86.5° than Spiro-OMeTAD (77.5°) (Fig. S11), which may be due to the more compact film of SF-MPA-MCz considering the same surface groups of both HTMs. Encouragingly, such uniform and dense film morphology of SF-MPA-MCz can facilitate hole mobility, as shown in space charge-limited current (SCLC) measurements (Fig. S12B). The hole mobility of pristine SF-MPA-MCz film is evaluated to be 4.5 × 10^−5^ cm^2^ V^−1^ s^−1^, higher than Spiro-OMeTAD (2.1 × 10^−5^ cm^2^ V^−1^ s^−1^). Accordingly, the trap-state densities of the hole-only devices based on Spiro-OMeTAD and SF-MPA-MCz were estimated to be 1.32 × 10^17^ cm^−3^ and 1.005 × 10^17^ cm^−3^, respectively (Fig. S12A). The reduced trap-state density of SF-MPA-MCz-based device indicates that SF-MPA-MCz can effectively passivate the defects on the perovskite surface, thus reducing the recombination losses during charge transfer.

### Theoretical studies

To have an in-depth insight into the varied electronic properties and intermolecular interactions of two HTMs, the theoretical calculations based on density functional theory (DFT) and time-dependent DFT (TD-DFT) were performed at the B3LYP/6-31 (d) level. As exhibited in Fig. S13, the HOMOs of Spiro-OMeTAD are delocalized over the whole molecule, and the lowest unoccupied molecular orbitals (LUMOs) are mainly located on the spirobifluorene core, consistent with the previous report [23]. By contrast, the discrete energy level distributions can be found for SF-MPA-MCz, with the HOMOs concentrated at the half of spirobifluorene having flanking diphenylamine units and the LUMOs distributed on the other half of spirobifluorene (Fig. 2F and G). We assume that the distinct energy level distributions of two HTMs originated from the anisotropic regulation strategy established here. The calculated HOMO/LUMO energy levels of Spiro-OMeTAD and SF-MPA-MCz are −5.43/0.09 eV and −5.57/−0.36 eV (Table S3), respectively, which are in good agreement with the experimental results. The images of the electrostatic potential surface (EPS) show that dominant-negative charges are concentrated on carbazole units as well as the substituted methoxy groups for SF-MPA-MCz (Fig. 2H), which may promote the electronic interactions between HTM and perovskite layer as will be confirmed later. The calculated exciton binding energy for SF-MPA-MCz (1.59 eV) is lower than Spiro-OMeTAD (1.88 eV), suggesting that electron-hole pairs are easier to dissociate into hole carriers for SF-MPA-MCz [28].

To clarify the intermolecular noncovalent interactions in the solid state, four types of dimers from the single-crystal structure of each HTM were extracted and not constructed arbitrarily (Fig. S14). By quantifying the total interaction energy (*E*_int_) of each dimer and adding them up, we found that SF-MPA-MCz possesses stronger intermolecular interactions than Spiro-OMeTAD, as observed in the ultraviolet–visible (UV–vis) absorption testing. Because of single-crystal structures, we further calculate the hole-transfer integral (*V*) and reorganization energy (*λ*) based on the same four dimers to provide insights into the intrinsic hole mobilities of HTMs. As depicted in Fig. S15 and Table S3, the total transfer integral of SF-MPA-MCz (31.83 meV) is twofold higher than Spiro-OMeTAD (15.68 meV), and the λ value of SF-MPA-MCz (400 meV) is smaller than Spiro-OMeTAD (410 meV). This indicates that SF-MPA-MCz features a higher hole transport capacity than Spiro-OMeTAD, which has been confirmed by the SCLC results.

Importantly, the interfacial interactions between HTMs and perovskite were also simulated by docking each HTM on the model of the perovskite surface (Fig. S16). Clearly, SF-MPA-MCz shows strong interfacial coupling with the perovskite surface via two methoxy groups on the rigid carbazole unit. In contrast, only one methoxy group of Spiro-OMeTAD can interact with perovskite due to the flexible nature of the diphenylamine unit. Therefore, the adsorption energy of SF-MPA-MCz (0.76 kcal/mol) was calculated to be higher than Spiro-OMeTAD (0.3 kcal/mol), which may be beneficial for underpinning interfacial adhesion and defect passivation as will be confirmed later.

### Photovoltaic performance and interfacial properties

**Fig. 3.**
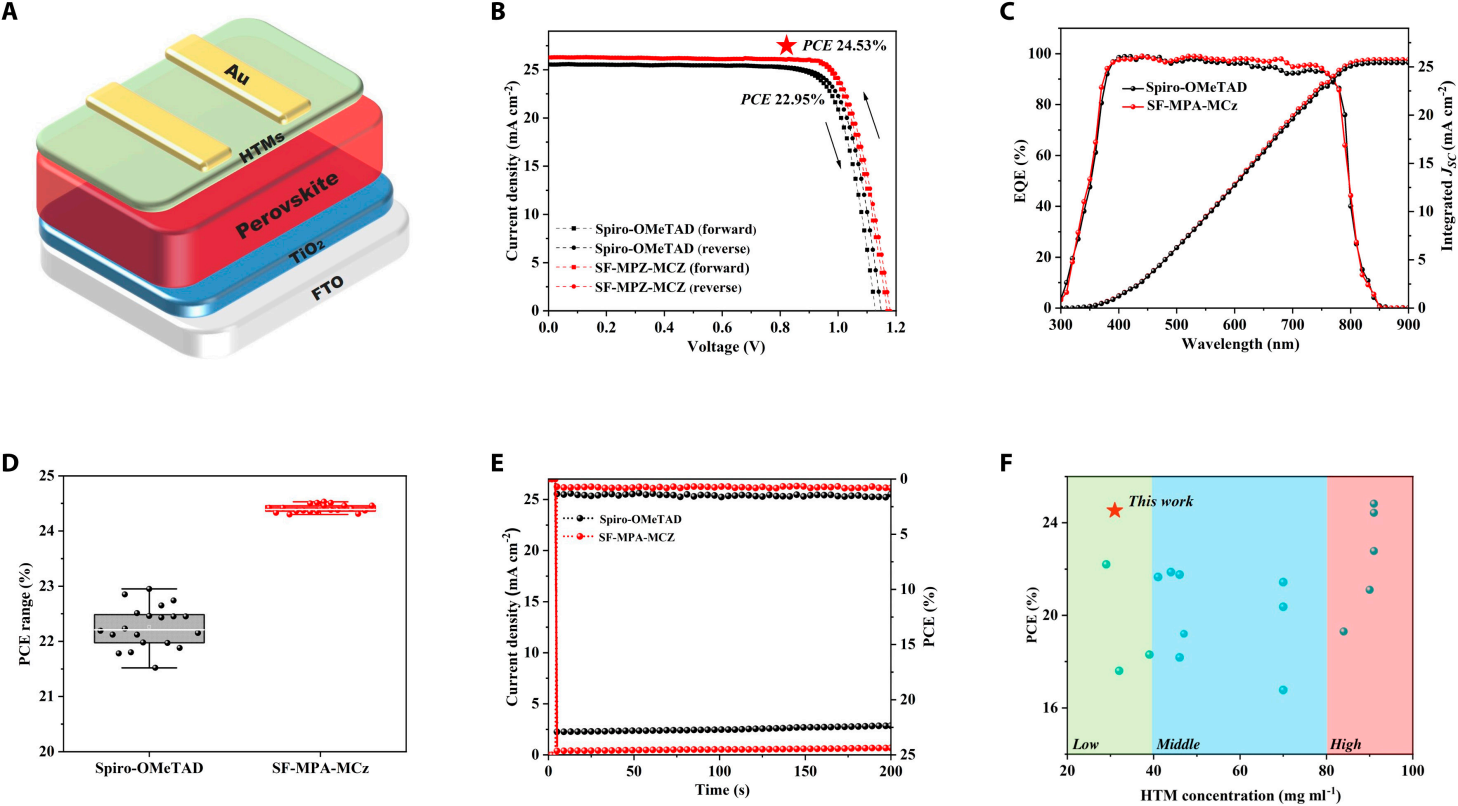
PSC performance. (A) Device structure in this work. (B) *J–V* curves and (C) EQE of the champion devices of PSCs based on different HTMs. (D) PCE statistical diagrams of (20 cells) devices with different HTMs. (E) MPP tracking for PSCs under continuous illumination. (F) Scatterplot of concentration efficiency with different spiro-type HTMs.

To explore the potential of de novo designed HTM SF-MPA-MCz in PSCs, the classical device configuration of FTO/TiO_2_/PVK/HTM/Au was adopted using Spiro-OMeTAD as control (Fig. 3A). After a series of optimization conditions, the SF-MPA-MCz-based device achieved a remarkably high PCE of 24.53% with *J*_SC_ of 26.24 mA cm^−2^, *V*_OC_ of 1.18 V, and *FF* of 79.22%, showing an all-round enhancement in photovoltaic parameters compared to 22.95% PCE of Spiro-OMeTAD-based device (*J*_SC_ of 25.51 mA cm^−2^, *V*_OC_ of 1.15 V, and *FF* of 78.2%) (Table 2). Moreover, SF-MPA-MCz shows a smaller hysteresis index and better device reproducibility than Spiro-OMeTAD (Fig. 3B). To accurately compare the PCE differences between SF-MPA-MCz and Spiro-OMeTAD-based PSCs (20 cells), the *Z* score was calculated according to the equation *Z = (η*_*1*_* − η*_*0*_*)/(σ*_*1*_*/*$\sqrt{N}$). The obtained *Z* score is 141.28, accompanied by a *P* value far less than 0.001, which means that the difference of PCE based on each HTM is extremely significant as presented in (Fig. 3D), and the corresponding photovoltaic parameters (*V*_OC_, *J*_SC_, *FF*) also exhibit all-round enhancement as presented in Fig. S17. The external quantum efficiency (EQE) spectra shown in Fig. 3C suggest that the integrated photocurrent densities (25.41 and 25.73 mA cm^−2^ for Spiro-OMeTAD and SF-MPA-MCz, respectively) agree well with those shown in the *J*–*V* curves. To confirm the accuracy of device PCEs, the maximum power point (MPP) tracking measurement was implemented (Fig. 3E). The SF-MPA-MCz-based device features a steady-state photocurrent and a stabilized power output for 200 s in MPP tracking, showing a stabilized efficiency of 24.37%, higher than that of Spiro-OMeTAD (22.36%). Notably, during device fabrication, the optimal concentration for SF-MPA-MCz solution is only 30 mg/ml, significantly lower than Spiro-OMeTAD (>70 mg/ml). Accordingly, the thickness of the SF-MPA-MCz layer is much reduced, as seen in cross-sectional scanning electron microscopy (SEM) (Fig. S18). It is delightful that a thin HTL can still enable high-performance n-i-p PSCs based on spiro-type HTMs due to the superior film morphology of SF-MPA-MCz (Fig. 3F). This is attractive because the material consumption and preparation cost can be remarkably reduced, especially for large-area processing in future commercialization (Table S4).

**Table 2.** The photovoltaic parameters of champion PSCs based on Spiro-OMeTAD and SF-MPA-MCz

Compounds			*J*_SC_ (mA cm^−2^)	*V*_OC_ (V)	* FF*	PCE_(MAX)_ (%)	PCE_(AVG)_ (%) ^a^
Spiro-OMeTAD	Forward	25.50	1.13	77.96	22.47	22.262 ± 0.381
	Reverse	25.51	1.15	78.20	22.95	
SF-MPA-MCz	Forward	26.24	1.17	79.10	24.28	24.409 ± 0.07
	Reverse	26.24	1.18	79.22	24.53	

^a^ Average PCE is obtained from 20 PSC devices.

**Fig. 4.**
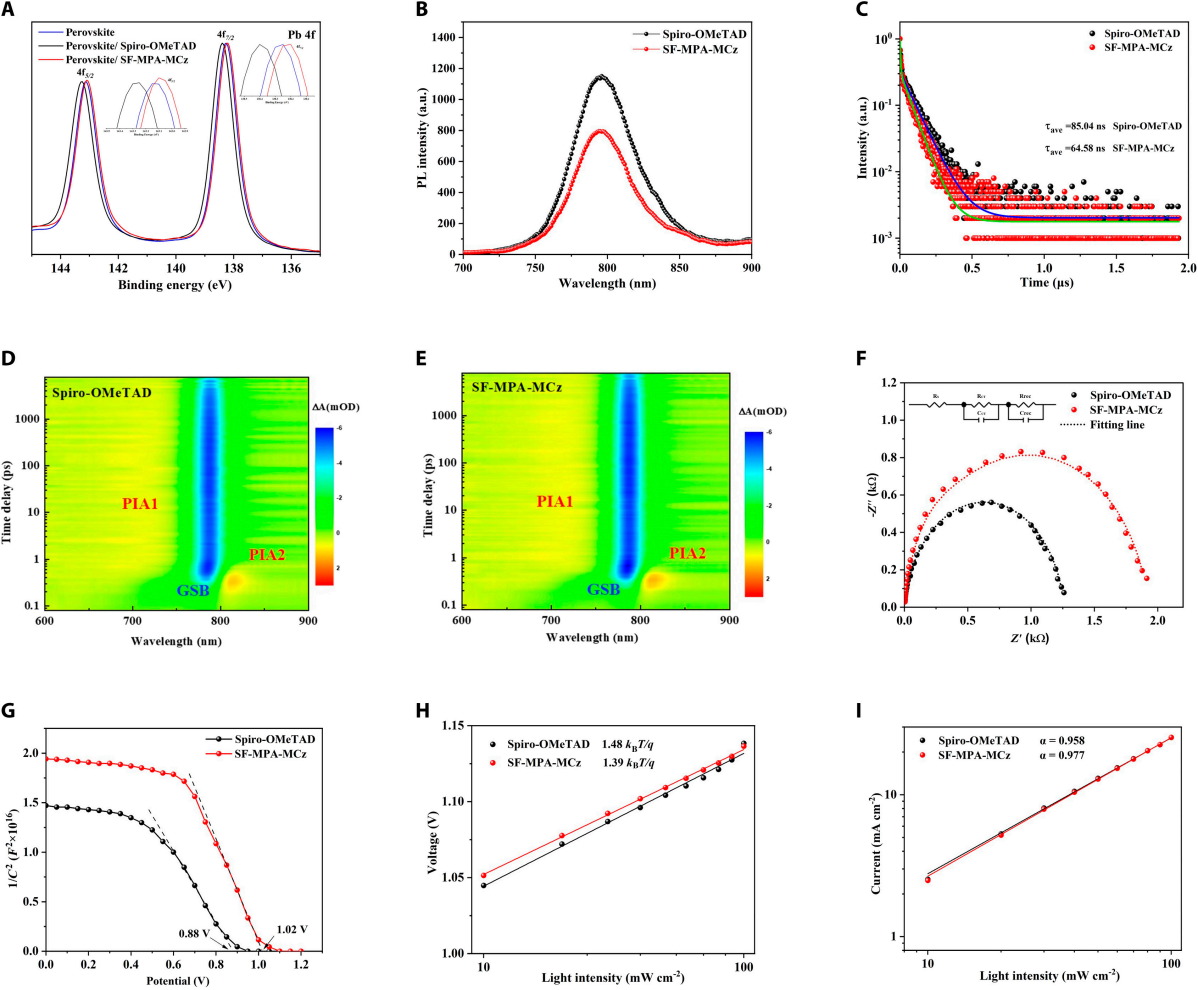
Interfacial properties and carrier dynamic characterization. (A) Pb 4f XPS spectra of pristine perovskite and different HTM-treated perovskite films. (B) Steady-state PL spectrum. (C) Time-resolved PL spectra of perovskite films coated with different HTMs. (D and E) Pseudo-contour plots with different HTM-treated perovskite films. (F) Nyquist plots of the impedance spectra with different HTMs. (G) Mott–Schottky plots of different HTMs. Dependence of (H) *V*_OC_ and (I) *J*_SC_ on light intensity.

To reveal the distinct interfacial properties brought by different HTMs, x-ray photoelectron spectroscopy (XPS) was first carried out, as shown in Fig. 4A. Interestingly, compared to pristine perovskite film with two main Pb 4f peaks at 138.25 and 143.15 eV, the perovskite films coated with two HTMs show different shift directions, i.e.*,* both peaks shift to higher binding energy for Spiro-OMeTAD, while to the lower binding energy for SF-MPA-MCz. Correspondingly, the O 1s peaks shift to lower binding energy for Spiro-OMeTAD after coating on perovskite, while to the higher binding energy for SF-MPA-MCz (Fig. S19). It is reasonable that SF-MPA-MCz may exhibit a stronger passivation effect toward uncoordinated Pb^2+^ ions via methoxy groups as evidenced by theoretical calculations, and different electronic states or compact lattice volumes are probably formed for Spiro-OMeTAD/perovskite interface [29,30]. Further, steady-state photoluminescence (PL) and time-resolved PL (TRPL) spectra (Fig. 4C) of the perovskites coated with Spiro-OMeTAD and SF-MPA-MCz were measured. As shown in Fig. 4B, SF-MPA-MCz quenched the PL of perovskite more effectively than Spiro-OMeTAD, implying better hole extraction ability. Also, PL decay time (*τ*) derived from TRPL for SF-MPA-MCz-treated perovskite (64.58 ns) is much shortened compared to Spiro-OMeTAD (85.04 ns), which further confirms the more efficient hole extraction and transfer at the perovskite/SF-MPA-MCz interface. The more robust evidence of photogenerated carrier dynamics is further provided by femtosecond transient absorption spectroscopy (fs-TAS). Figure 4D and E displays the pseudo-color fs-TAS plots of samples as a function of delay time and wavelength. The main photobleaching negative peak at ~780 nm for all samples can be observed clearly, which is attributed to the state-filling of the carriers at the band edge. Two positive peaks (PIA1 and PIA2) represent the photoinduced absorption. The transient absorption (TA) decay kinetics shown in Fig. S20A and B exhibit a faster decrease of *ΔA* in the sample with SF-MPA-MCz than that with Spiro-OMeTAD, indicating more effective carrier separation/extraction in the sample with SF-MPA-MCz. Subsequently, electrochemical impedance spectroscopy (EIS) was employed to investigate the charge recombination behavior in PSCs. The Nyquist plots are shown in Fig. 4F, and the corresponding parameters are summarized in Table S5. The lower sheet resistance (*R*_s_) and charge transfer resistance (*R*_CT_) values of SF-MPA-MCz indicate its better hole transport and extraction capacity, while the higher recombination resistance (*R*_rec_) of SF-MPA-MCz (1,962 Ω) compared to Spiro-OMeTAD (1,258 Ω) means the more effectively suppressed defect-assisted nonradiative recombination in SF-MPA-MCz-based device. Mott–Schottky analysis further reveals that the built-in potential (*V*_bi_) is estimated to be 0.88 and 1.02 V for the Spiro-OMeTAD- and SF-MPA-MCz-based devices, respectively (Fig. 4G). The higher *V*_bi_ of the SF-MPA-MCz-based device may provide a strong driving force for carrier separation and transport, which is beneficial for inhibiting interfacial charge recombination. To gain deeper insight into the charge recombination kinetics, the dependence of the *J*_SC_ and *V*_OC_ of PSCs on light intensity (*I*_light_) is performed. As shown in Fig. 4H, the SF-MPA-MCz-based device exhibits a smaller slope value (1.39 *K*_B_*T*/*q*) of *V*_OC_ versus *I*_light_ curves than that of Spiro-OMeTAD (1.48 *K*_B_*T*/*q*). As for *J*_SC_ versus *I*_light_ curves in Fig. 4I, which is fitted by a power–law equation (*J*_SC_ ∝ *I*_light_^α^), SF-MPA-MCz-based device possesses a higher α value of 0.977 than Spiro-OMeTAD (0.958). These results imply that charge recombination at interfaces can be efficiently suppressed for SF-MPA-MCz-based PSCs [31].

### Stability assessment

**Fig. 5.**
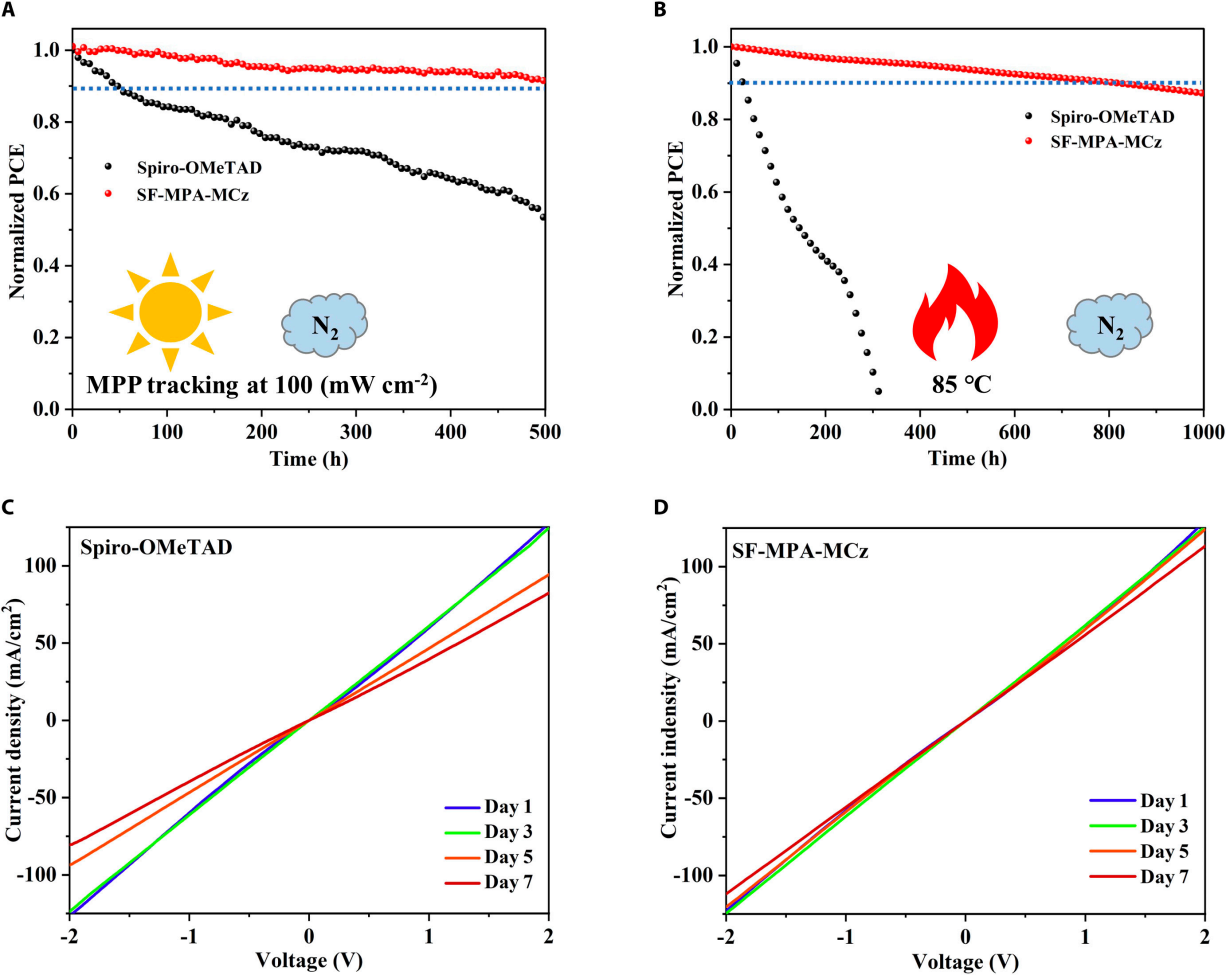
Operating stability. (A) Time-dependent light soaking stability tests of the PSCs under continuous 1 sun illumination at their MPP. (B) Time-dependent thermal stability tests of the PSCs at 85 °C in N_2_. (C and D) Conductivity of Spiro-OMeTAD and SF-MPA-MCz under light soaking and heating treatment.

Excellent operational stability is essential for the commercial application of PSCs. Figure 5A shows the full sunlight soaking age under the continuous MPP (100 mW cm^−2^) tracking. It can be seen that the SF-MPA-MCz-based unencapsulated perovskite device can maintain more than 90% PCE after 500 h of lasting test, while the PCE of the Spiro-OMeTAD-based unencapsulated PSCs drops to about ~50% under the same conditions. Further, the devices were placed in a glove box to monitor their thermal stability at 85 °C. As shown in Fig. 5B, the Spiro-OMeTAD-based unencapsulated device cannot work after heating for 300 h, but more than 85% PCE can be maintained after 1,000 h of heating for SF-MPA-MCz-based counterpart. We assume that significantly enhanced device stability is ascribed to the stability improvement of SF-MPA-MCz HTL, considering the same device structure and other functional layers in the control experiments. To confirm this, we first measured the conductivity of each HTM in a pristine film state using linear sweep voltammetry (LSV). As shown in Fig. S21, after 1 week of continuous light soaking, both HTMs exhibit a tiny decline in conductivity. However, when the external conditions became harsh, i.e., treating with both light soaking and heat aging, the conductivity of Spiro-OMeTAD is severely reduced, while SF-MPA-MCz maintains good conductivity (Fig. 5C and D). This indicates that the enhanced thermal stability of SF-MPA-MCz is propitious for maintaining good electrical properties under high temperatures so that high efficiency can persist during long-term device operation.

Furthermore, SF-MPA-MCz can protect the perovskite layer more effectively from external stresses, as shown in Fig. S22. After light soaking and heating aging, we compared the fresh perovskite films with those coated with HTMs. For a clear comparison, the HTLs were carefully washed off with chlorobenzene (CB) after aging tests. Evidently, the perovskite coated with Spiro-OMeTAD suffers obvious degradation after light soaking for 7 days, which becomes much more severe after exposure to continuous light soaking at 80 °C (Fig. S22E and G). In contrast, the perovskite treated with SF-MPA-MCz shows nearly no change under light soaking unless the heating aging is additionally involved (Fig. S22H and F).

To explain the enhanced device performance and stability based on SF-MPA-MCz, we present a compilation of reasons as follows: (a) By introducing rigid carbazole units, the thermal stability of SF-MPA-MCz is remarkably improved to resist heat aging and maintain film morphology so that the operational lifetime can be prolonged. (b) The planar structure of carbazole units can strengthen intermolecular stacking and contribute to pretty compact and uniform film morphology, which endows high hole mobility, good hydrophobicity, and preferable surface coverage with SF-MPA-MCz. This is also preferable for reducing material consumption during the solution process. (c) Apart from the molecular arrangement in the bulk film of HTMs, the anisotropic regulation toward SF-MPA-MCz can significantly affect the interfacial properties. Due to the better energy level alignment and more effective passivation effect of SF-MPA-MCz compared to Spiro-OMeTAD, the defects that existed on the perovskite surface can be mitigated, and the hole extraction can be enhanced so that interfacial charge recombination can be inhibited to boost device efficiency further. Collectively, it is anticipated that such an anisotropic regulation strategy may render diverse molecular design to traditional spiro-type HTMs as many widely used building blocks (e.g., phenoxazine, phenothiazine, and 5*H*-pyrido[4,3-b]indole) can be combined and introduced selectively at the single molecular level, which is applausive for nontrivial manipulation of electrical and film morphological properties of HTMs. The corresponding work is underway in our laboratory, and exploring more efficient and stable HTMs is highly promising.

## Discussion

In summary, we put forward an anisotropic regulation strategy in this work and designed and synthesized a novel HTM SF-MPA-MCz, which features diphenylamine and carbazole moieties on different parts of the spiro-skeleton. Compared to Spiro-OMeTAD, introducing rigid and planar carbazole units at the single molecular level can enhance the structural thermal stability, lower HOMO energy level, and optimize film morphology. The uniform and dense film endows SF-MPA-MCz with high hole mobility, hydrophobicity, and superior interfacial contact. Also, the compact and stable HTL film morphology can reduce material dosage during the solution process and prolong operational stability despite using dopants. As a result, an appreciably high PCE of 24.53% was achieved for SF-MPA-MCz-based PSC, much surpassing that of Spiro-OMeTAD (22.95%). More importantly, high stability can be realized for SF-MPA-MCz-based devices with over 90% PCE remaining under 500-h continuous light soaking and over 85% PCE maintained under 1,000-h heating at 85 °C. This work establishes a new design idea to enhance the flexibility and diversity of the reconstruction of HTMs. We believe that this anisotropic regulation strategy may facilitate the exploration of more efficient and stable HTMs to replace Spiro-OMeTAD, and the corresponding work is undergoing in our laboratory.

## Materials and Methods

### Materials

F-doped tin oxide (FTO) glass substrate (8 Ω per square) was purchased from Suzhou Shangyang Solar Energy Technology Co. Ltd. Formamidine hydroiodate (FAI), methyl ammonium iodide (MAI), cesium iodide (CsI), methyl ammonium chloride (MACl), and 2,2′,7,7′-tetrakis(*N*,*N*-di-p-methoxyphenylamine)-9,9′-spirobifluorene (spiro-MeOTAD) were purchased from Liaoning Optimum New Energy Technology Co. Ltd. Lead iodide (PbI_2_), lead bromide (PbBr_2_), and isopropanol (IPA) were purchased from TCI. Phenethylmethoxy iodide (PEAI), bistrifluoromethanesulfonimide lithium salt (Li-TFSI), 4-tert-butylpyridine (t-BP), and tris(2-(1*H*-pyrazol-1-yl)-4-tert-butylpyridine)-cobalt(III)-tris(bis-(trifluoromethylsulfonyl)imide) (FK-209) were purchased from Xi’an Yuri Solar Co. Ltd. Titanium tetrachloride (TiCl_4_), dimethylformamide (DMF), dimethyl sulfoxide (DMSO), acetonitrile (ACN), and chlorobenzene (CB) were purchased from Sigma-Aldrich.

### Solution preparation

Precursor solution for perovskite films was prepared by dissolving 1.73 M PbI_2_, 1.47 M FAI, 0.173 M MAI, 0.0519 M PbBr_2_, 0.0865 M C_S_I, and 0.268 M MACl in DMF/DMSO mixed solvent (v:v = 5:1). The precursor solution was stirred for 4 h at room temperature in the glove box and was filtered before use. To prepare Spiro-OMeTAD solution, 73 mg of Spiro-OMeTAD, 28 μl of t-BP, 20 μl of Li-TFSI (520 mg/ml in ACN), and 20 μl of FK-209 (300 mg/ml in ACN) were added into 1 ml of CB. SF-MPA-MCz solution in CB (30 mg ml^−1^) was mixed with 15 μl of t-BP, 10 μl of Li-TFSI solution (520 mg/ml in ACN), and 10 μl of FK-209 (300 mg/ml in ACN).

### Device fabrication

The FTO glass substrates were cleaned with deionized water, acetone, and ethanol sequentially in a sonication bath for 25 min. To prepare the TiO_2_ film, first, the FTO glass substrate was treated in a UV-ozone cleaner for 20 min, and the treated FTO glass substrate was deposited in a water bath at 90 °C for 50 min by chemical water bath method using TiCl_4_/deionized water solution (3 ml/150 ml). Then, the TiO_2_ film substrates were transferred to the glove box. For the deposition of the perovskite layer, the perovskite precursor solutions were spin-coated on the FTO/TiO_2_ substrate in a two-step program at 1,500 rpm for 4 s and 5,000 rpm for 20 s. During the two-step spin-coating procedure, 900 μl of diethyl ether was quickly dropped at 10 s prior to the end of the whole program. The substrates were then annealed at 150 °C for 15 min. After the perovskite film was cooled down to room temperature, 60 μl of PEAI solution (5 mg/ml in IPA) was spin-coated at 5,500 rpm for 20 s. Then, the 40-μl HTL solution was spin-coated on the FTO/TiO_2_/PVK/PEAI substrates at 4,000 rpm for 30 s. Finally, the ~100-nm gold layer was evaporated under high vacuum.

### Characterization

The photocurrent density–voltage (*J*–*V*) curves of devices were measured with a Keithley 2400 source meter using a solar simulator (Amazing Light SS-F5-3A) at AM 1.5G illumination (100 mW cm^−2^) equipped with a calibrated Si reference cell. The surface morphologies and cross-sectional structure of these perovskite films were investigated using SEM (HITACHI Regulus SU8100). XRD patterns of the films were obtained by x-ray diffractometer (UItima IV X-ray diffractometer) with Cu Kα radiation. AFM spectra were measured by Dimension Icon (Bruker AXS) at tapping mode. The electrochemical CV was performed in a 0.1 M tetrabutylammonium hexafluorophosphate (Bu_4_NPF_6_) chloroform solution as the supporting electrolyte with a scan speed at 0.05 V/s. A ferrocene/ferrocenium redox couple was used as an external standard. A Pt wire, glassy carbon discs, and Ag/AgCl were used as the counter, working, and reference electrodes, respectively. The UPS spectra were collected on the X-ray Photoelectron Spectrometer (Thermo Fisher Scientific, ESCALAB 250Xi, UK). UV–vis spectra were measured with a PerkinElmer Lambda 950. The PL spectrum and TRPL signals of the film were measured by using an Edinburgh FLS1000 spectrophotometer. EIS measurements were obtained on an electrochemical workstation (Germany, Zahner Company) with a frequency range from 80 Hz to 5 MHz in the dark. The space-charge limited current (SCLC) of devices was measured by a Keithley 2400 source meter in the dark. GIWAXS experiments were conducted at beamline 7.3.3 at the Advanced Light Source at Lawrence Berkeley National Lab (Berkeley, USA). The samples were illuminated with 10-keV radiation (λ = 1.24 Å) at an incident angle (α) of 0.12°. The beam size was 300 μm (height) × 700 μm (width). The scattering signal was captured on a Pilatus 2M (172-μm pixel size, file format EDF, 1,475 × 1,679 pixels) located 282 mm from the sample. Acquisition times were 10 s for each frame.

## Data Availability

All data needed to evaluate the conclusions in the paper are present in the paper and/or the Supplementary Materials.

## Supplementary Materials

Supplementary 1 Experimental Section Figs. S1 to S22 Tables S1 to S5
