# Supplementary material for: De Novo Design of Spiro-Type Hole-Transporting Material: Anisotropic Regulation Toward Efficient and Stable Perovskite Solar Cells
Source: Research (Wash D C). 2024 Mar 23;7:0332. doi: 10.34133/research.0332 (PMC10964223; doi:10.34133/research.0332)
Supplement: Supplementary 1 — Experimental Section Figs. S1 to S22 Tables S1 to S5 [file research.0332.f1.docx]

Supplementary Materials

**De novo Design of Spiro-type Hole-transporting Material: Anisotropic Regulation toward Efficient and Stable Perovskite Solar Cells**

Xu-Ran Wang et al.

*Corresponding author. Email: q397983012@126.com; peng.gao@fjirsm.ac.cn; ifewangy@fjnu.edu.cn; vc@nwpu.edu.cn

**This file includes:**

Figs. S1 to S22; Table S1 to S5; Molecular synthesis, characterization and theoretical calculation methods.

**Experimental Section**

**Materials and synthesis**

All reagents and chemicals are commercially purchased and directly used without further purification unless otherwise stated. Solvents were purified by standard methods and dried if necessary. The starting material (2,7-dibromo-2’,7’-diiodospirobifluorene) is prepared by referencing the method reported by Tsutsui et al (1).


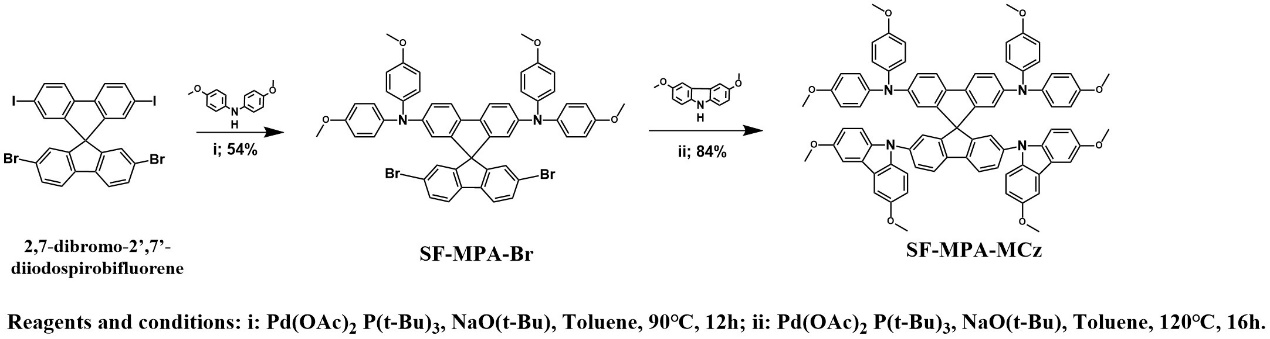


**Scheme S1.** Synthetic route for SF-MPA-MCz

**Synthetic**

2',7'-dibromo-N2,N2,N7,N7-tetrakis(4-methoxyphenyl)-9,9'-spirobi[fluorene]-2,7-diamine (**SF-MPA-Br**): To a two necked flask, 2,7-dibromo-2’,7’-diiodospirobifluorene (150 mg, 0.2 mmol), bis(4-methoxyphenyl)amine (92 mg, 0.4 mmol), palladium (II) acetate (Pd(OAc)_2_) (1 mg, 0.004 mmol), tri-tert-butylphosphine (P(t-Bu)_3_) (20 μL, 0.008 mmol) and sodium tert-butoxide (NaO(t-Bu)) (58 mg, 0.6 mmol) were added in order, and then argon replacement three times, toluene (5 mL) was added. The mixture was stirred at 90 ℃ for 12 h. After cooling to room temperature, the solvent was removed under reduced pressure, the residue was poured into water and extracted with dichloromethane (DCM), and then the organic phase was dried over Na_2_SO_4_. The organics were concentrated by evaporation, and the crude product was purified by column chromatography (petroleum ether (PE): DCM = 1:1). Pale yellow solid 103 mg obtained with a yield of 54%.^1^H NMR (500 MHz, Methylene Chloride-d_2_) δ 8.05 (d, J = 7.9 Hz, 2H), 7.43 (dd, J = 8.0, 2.0 Hz, 2H), 7.04 (dd, J = 7.5, 1.8 Hz, 4H), 6.99 (d, J = 1.9 Hz, 2H), 6.92 – 6.85 (m, 10H), 6.73 (m, 8H), 6.20 (d, J = 8.2 Hz, 4H), 3.77 (s, 12H). HRMS: C_53_H_40_Br_2_N_2_O_4_ calcd: 926.1355, found: 926.1359 [M^+^] (Fig. S1).

2',7'-bis(3,6-dimethoxy-9H-carbazol-9-yl)-N2,N2,N7,N7-tetrakis(4-methoxyphenyl)-9,9'-spirobi[fluorene]-2,7-diamine (**SF-MPA-MCz**): Synthesized in an analogue way as **SF-MPA-Br**. **SF-MPA-Br** (60 mg, 0.064 mmol), 3,6-dimethoxy-9H-carbazole (36 mg, 0.1536 mmol (2.4 eq)), Pd(OAc)_2_ (0.96 mg, 0.00384 mmol), P(t-Bu)_3_ (19.2 μL, 0.00768 mmol), NaO(t-Bu) (18 mg, 0.192 mmol) and toluene (5 mL). The mixture was stirred at 120℃ for 16 h. The crude product was purified by column chromatography PE: DCM = 1: 3). Pale yellow solid (66.2 mg) was obtained with a yield of 84%.^1^H NMR (400 MHz, DMSO-d_6_): δ 8.16 (d, J = 8.1 Hz, 2H), 7.83 (d, J = 2.4 Hz, 4H), 7.63-7.60 (m, 4H), 7.13 (d, J = 8.9 Hz, 4H), 7.01 (dd, J = 8.9, 2.4 Hz, 4H), 6.89-6.85 (m, 10H), 6.79 (dd, J = 8.4, 2.1 Hz, 2H), 6.73 (d, J = 9.0 Hz, 8H), 6.32 (d, J = 2.1 Hz, 2H), 3.88 (s, 12H), 3.65 (s, 12H) (Fig. S2). ^13^C NMR (151 MHz, Methylene Chloride-d2) δ 155.89, 154.54, 151.61, 149.16, 139.90, 137.88, 136.46, 126.45, 125.87, 123.94, 122.94, 122.41, 121.78, 120.30, 117.63, 115.44, 114.81, 110.89, 103.12, 56.32, 55.76 (Fig. S3). Elemental analysis: calcd: C, 79.65; H, 5.28; N, 4.59; found: C, 79.95; H, 5.16; N, 4.78. MALDI-TOF: C_81_H_64_N_4_O_8_ calcd: 1220.47242, found: 1220.5 [M^+^] (Fig. S4).

**Theoretical calculations**

Energy decomposition analysis (EDA) was carried out using an AMBER force field for dimers in single-crystals with the MultiWFN package (version 3.8) (*2*), also based on single-point energy calculation at the M06-2X/6-31G(d,p) level of theory. The total intermolecular interaction energy (*E^int^*) can be decomposed into the electrostatic (*E^ele^*), repulsion (*E^rep^*), and dispersion (*E^disp^*) energies as described by

***E^int^* = *E^ele^* + *E^rep^* + *E^disp^* (1)**

The hole-transfer integral (*V*) is defined as the strength of electronic coupling between the highest occupied molecular orbitals (HOMO) of molecules in dimers of single crystals by the equation

$\boldsymbol{V=}\left\langle\boldsymbol{\varphi}_{\boldsymbol{i}}^{\boldsymbol{HOMO}} \left| \boldsymbol{F} \right|\boldsymbol{\varphi}_{\boldsymbol{f}}^{\boldsymbol{HOMO}} \right\rangle$ **(2)**

Where 𝜑 represents the wavefunction, and *F* is the Fock operator. The hole-transfer integral was calculated with the Gaussian 09 program package at the M06-2X/6-31G(d,p) level of theory. The hole-transfer rate constant (*ki*) can be described by Marcus' theory as follows (*3*):

$\boldsymbol{k}_{\boldsymbol{i}}\boldsymbol{=}\frac{\boldsymbol{4}\boldsymbol{\pi}^{\boldsymbol{2}}\boldsymbol{V}_{\boldsymbol{i}}^{\boldsymbol{2}}}{\boldsymbol{h}}\frac{\boldsymbol{1}}{\sqrt{\boldsymbol{4}\boldsymbol{\pi\lambda}\boldsymbol{K}_{\boldsymbol{B}}\boldsymbol{T}}}\boldsymbol{exp}\left[ \boldsymbol{-}\frac{\boldsymbol{\lambda}}{\boldsymbol{4}\boldsymbol{K}_{\boldsymbol{B}}\boldsymbol{T}} \right]$ **(3)**

Where *Vi* is the hole-transfer integral between adjacent molecules in a single crystal structure, *λ* is the reorganization energy, *h* is the Planck constant, *k_B_* is the Boltzmann constant, and *T* is the temperature in kelvin. The reorganization energy (*λ*) of hole-transfer is composed of internal reorganization energy and external reorganization energy. The external part can be ignored because of its little contribution to the solid state. The internal part is dependent on the change of molecular geometry during the hole-transfer process. By definition:

$\boldsymbol{\lambda\approx}\boldsymbol{\lambda}_{\boldsymbol{int}}\boldsymbol{=}\left( \boldsymbol{E}_{\boldsymbol{0}}^{\boldsymbol{*}}\boldsymbol{+}\boldsymbol{E}_{\boldsymbol{0}} \right)\boldsymbol{+}\left( \boldsymbol{E}_{\boldsymbol{+}}\boldsymbol{-}\boldsymbol{E}_{\boldsymbol{+}}^{\boldsymbol{*}} \right)$ **(4)**

where $E_{0}^{*}$ is the energy of neutral in ion geometry, $E_{0}$ is the energy of neutral in neutral geometry, $E_{+}$ is the energy of ion in ion geometry, and $E_{+}^{*}$ is the energy of an ion in neutral geometry. The diffusion coefficient (*D*) is defined as:

$\boldsymbol{D=}\frac{\boldsymbol{1}}{\boldsymbol{2}\boldsymbol{n}}\sum_{\boldsymbol{i}} \boldsymbol{d}_{\boldsymbol{i}}^{\boldsymbol{2}}\boldsymbol{k}_{\boldsymbol{i}}\boldsymbol{p}_{\boldsymbol{i}}$ **(5)**

where *n* represents the dimensionality of the single crystal with a value of 3. *Di* and *ki* are the centroid distance and the hopping rate, respectively. *Pi* is the relative hopping probability via path i, which can be generalized as:
 $\boldsymbol{p}_{\boldsymbol{i}}\boldsymbol{=}\frac{\boldsymbol{k}_{\boldsymbol{i}}}{\sum_{\boldsymbol{i}} \boldsymbol{k}_{\boldsymbol{i}}}$ **(6)**

In the end, the charge mobility (*µ*) can be derived via the Einstein-Smoluchowski equation, where e is the elementary charge.

$\boldsymbol{\mu=}\frac{\boldsymbol{eD}}{\boldsymbol{k}_{\boldsymbol{B}}\boldsymbol{T}}$ **(7)**

The interaction energies (*ΔE*) calculations based on density functional theory (DFT) were performed using the Vienna ab initio simulation package (*4*) through the following formula:

$\boldsymbol{\Delta E=}\boldsymbol{E}_{\boldsymbol{inter}}\boldsymbol{-}\boldsymbol{E}_{\boldsymbol{htm}}\boldsymbol{-}\boldsymbol{E}_{\boldsymbol{perov}}$ **(8)**

Where *E*_𝑖𝑛𝑡𝑒𝑟_ is the energy of the interface, *E*_ℎ𝑡𝑚_ is the energy of the isolated molecules, and *E*_𝑝𝑒𝑟𝑜𝑣_ is the energy of the isolated perovskite slab. DFT calculations used the Projector Augmented Wave (PAW) method to describe the effects of core electrons and Perdew-Burke-Ernzerhof (PBE) (*5*) implementation of the Generalized Approximation (GGA) for the exchange-correlation functional. The energy cutoff for the plane-wave basis set of 500 eV was used for the calculations. The total energy tolerance for the electronic energy minimization was 10^-4^ eV; for structure optimization, forces were minimized such that all atoms experience forces < 0.05 eV Å^-1^ after relaxation of the ionic coordinates and unit cell shape and volume. The k-point meshes for different structures were generated according to the Gamma-centered scheme for the Brillouin zones. The perovskite model system comprises 3×3×1 perovskite slabs oriented along the 010 direction for the MAI-terminated surface.

For the calculations of adsorption energy between perovskite and HTMs, density functional theory (DFT) was used, and the Projector Augmented Wave (PAW) potential was implemented with the Device Studio (*6*), which provides a number of functions for performing visualization, modeling, and simulation. Molecular geometry simulation using BDF (*7*, *8*) software integrated in Device Studio program. We used the B3LYP function to calculate the molecules and optimized the molecular structure using the Def2-SVP basis set. For the calculation of the doping mechanism, DFT calculations were performed by using the DS-PAW (*9*) package in the Device Studio program. The generalized gradient approximation in the Perdew-Burke-Ernzerhof (PBE) format was used to compute exchange and correlation energies, and a plane wave basis set cutoff energy of 550 eV was adopted. Grimme’s DFT-D3 was used for dispersion correction. A mesh of 1×1×1 Monkhorst-Pack k-points was used for the Brillouin-zone integration. The criterion of electron self-consist iteration was set as 1.0 × 10^-5^ eV and the maximum force was relaxed down to 0.05 eV Å^-1^. VESTA (*10*) was used to analyze and visualize electronic structure data.

HZWTECH is thanked for allowing the use of the DS-PAW and BDF code in Device Studio. The authors also gratefully acknowledge HZWTECH for supporting the computational resources.


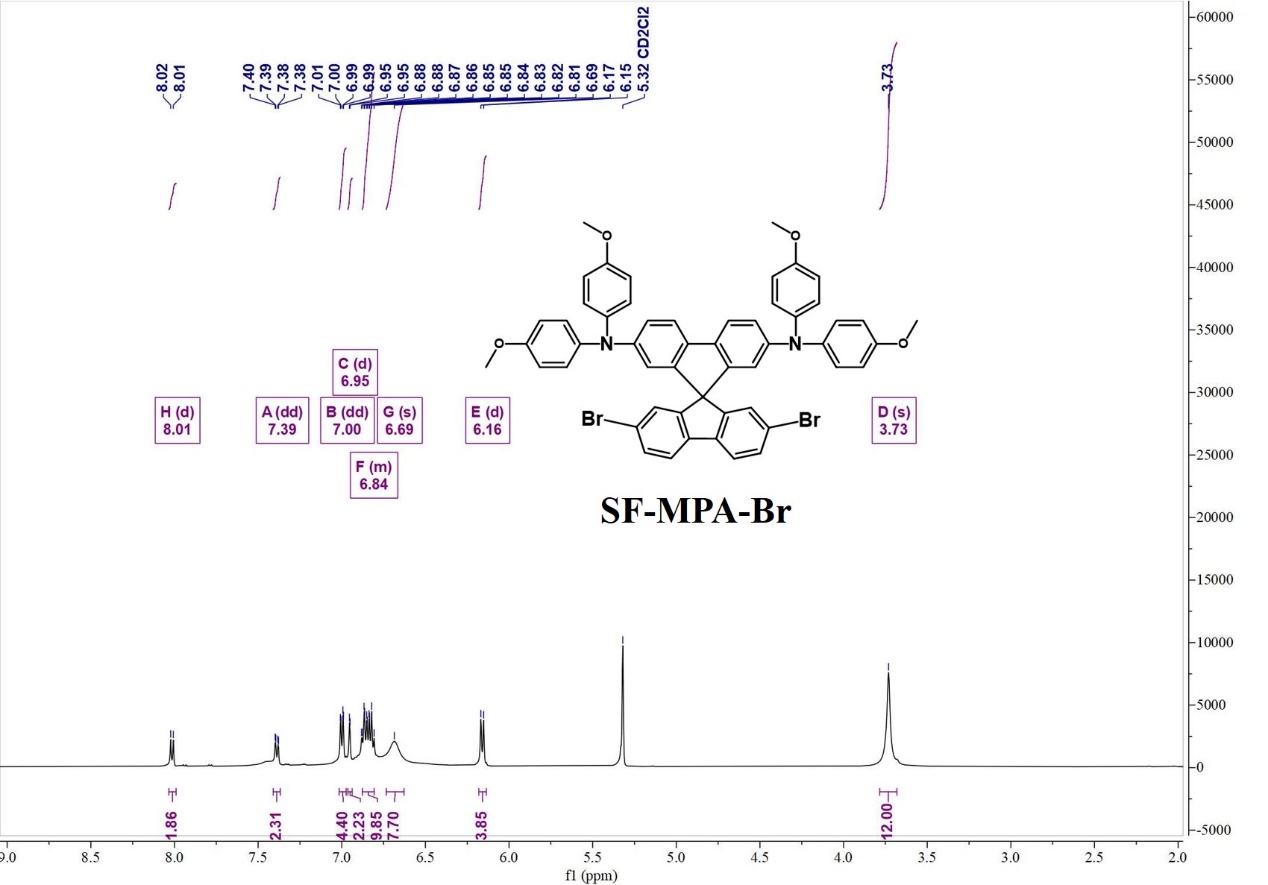


**Fig. S1.** The ^1^ H NMR spectrum of SF-MPA-Br.


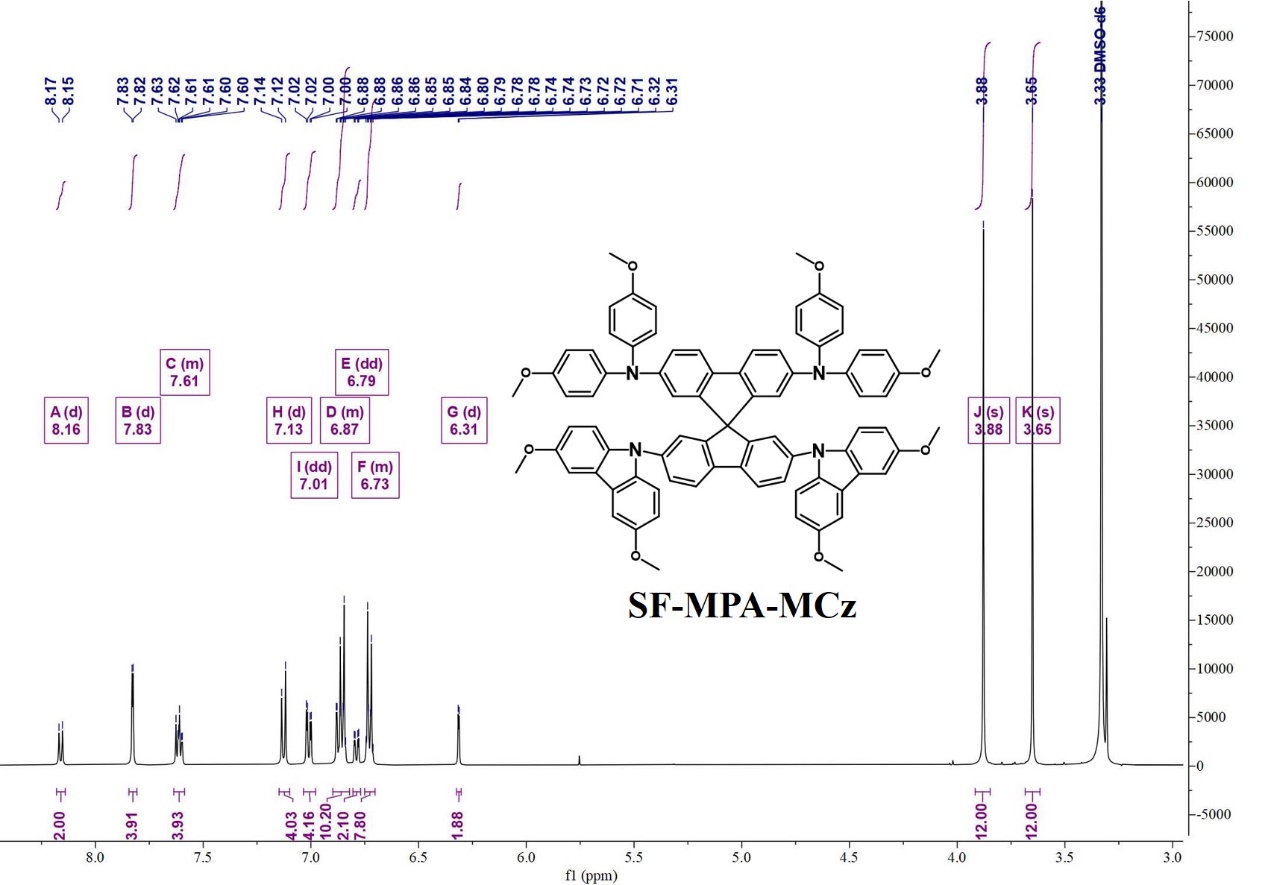


**Fig. S2.** The ^1^ H NMR spectrum of SF-MPA-MCz.


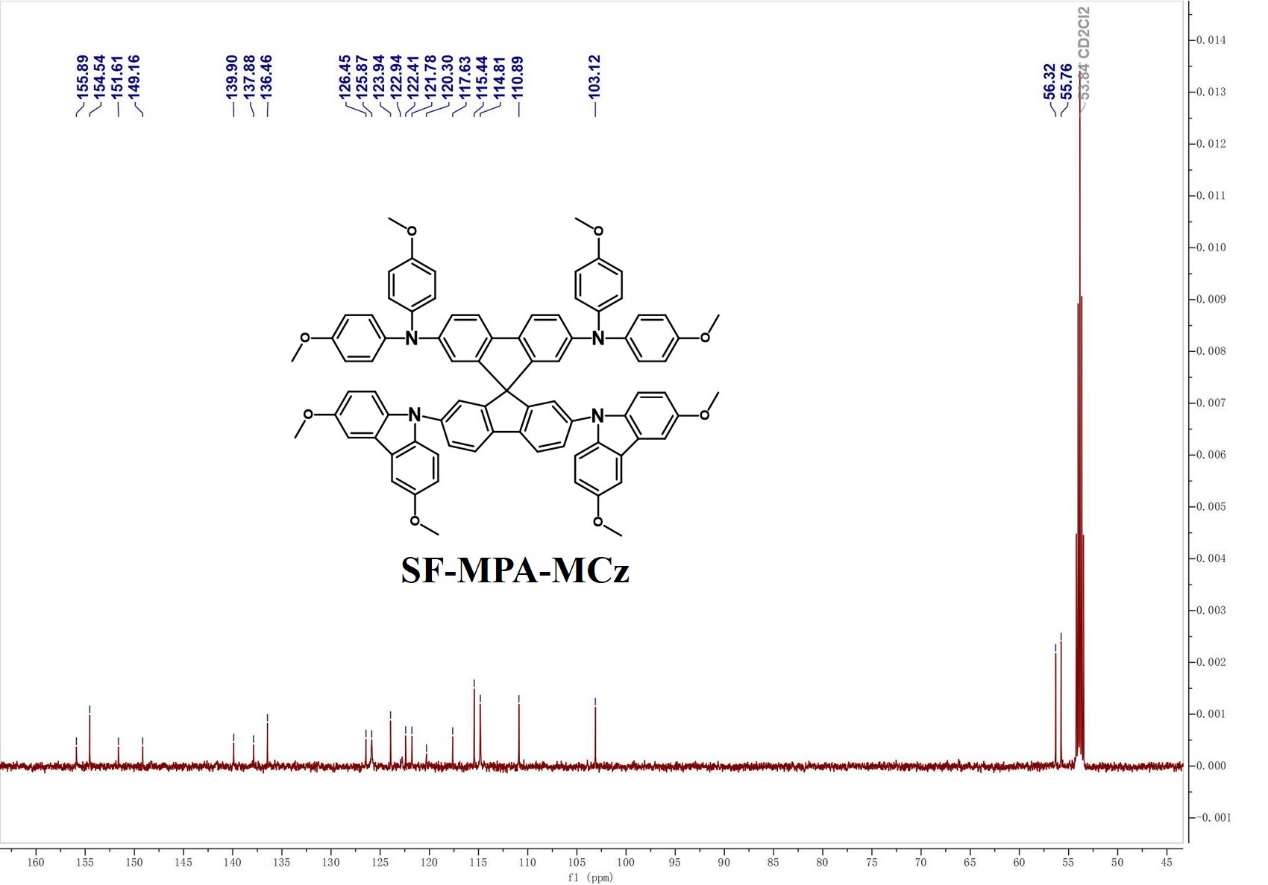


**Fig. S3.** The ^13^ C NMR spectrum of SF-MPA-MCz.


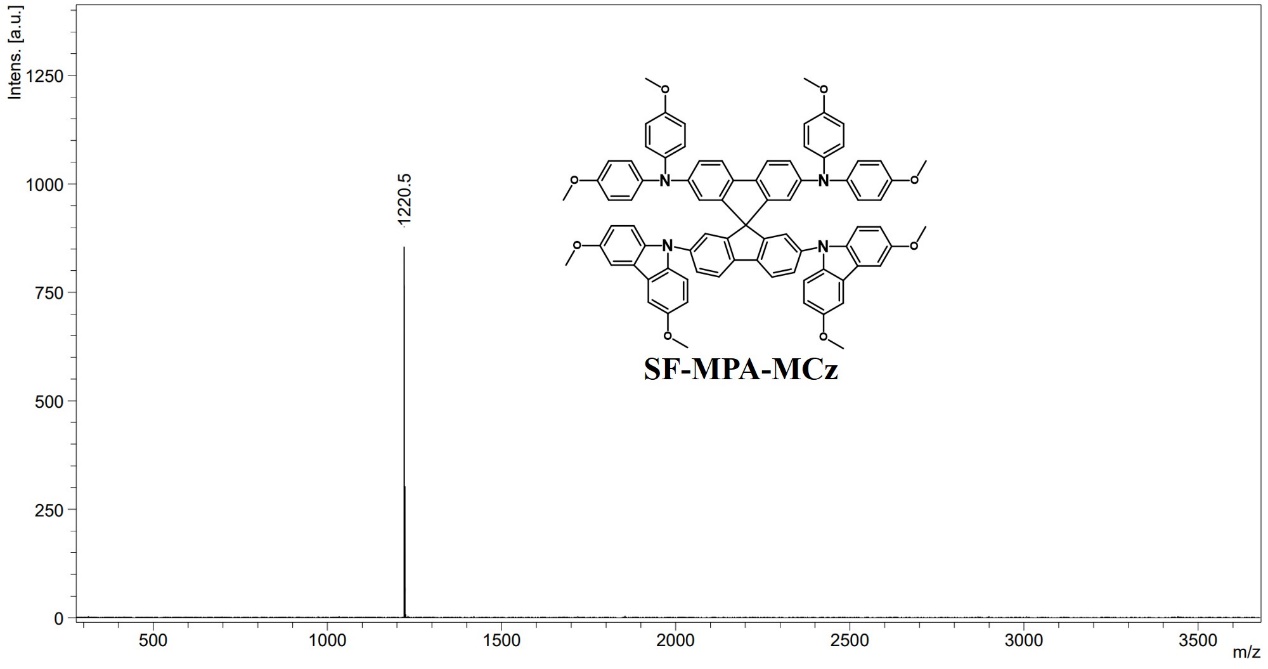


**Fig. S4.** MALDI-TOF of SF-MPA-MCz.


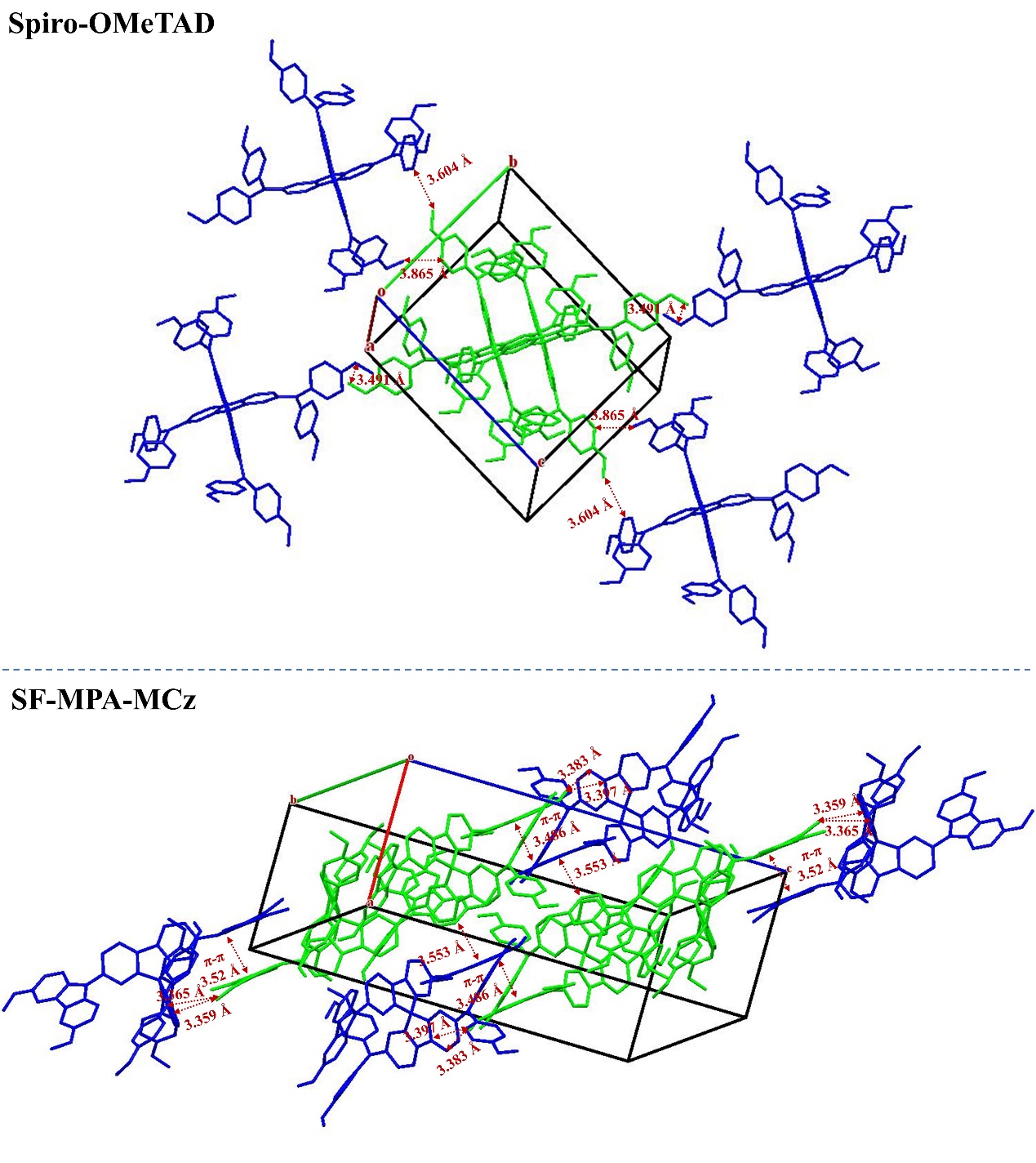


**Fig. S5.** Embedded molecules (blue) around the single-crystal unit cell (green) of Spiro-OMeTAD and SF-MPA-MCz.


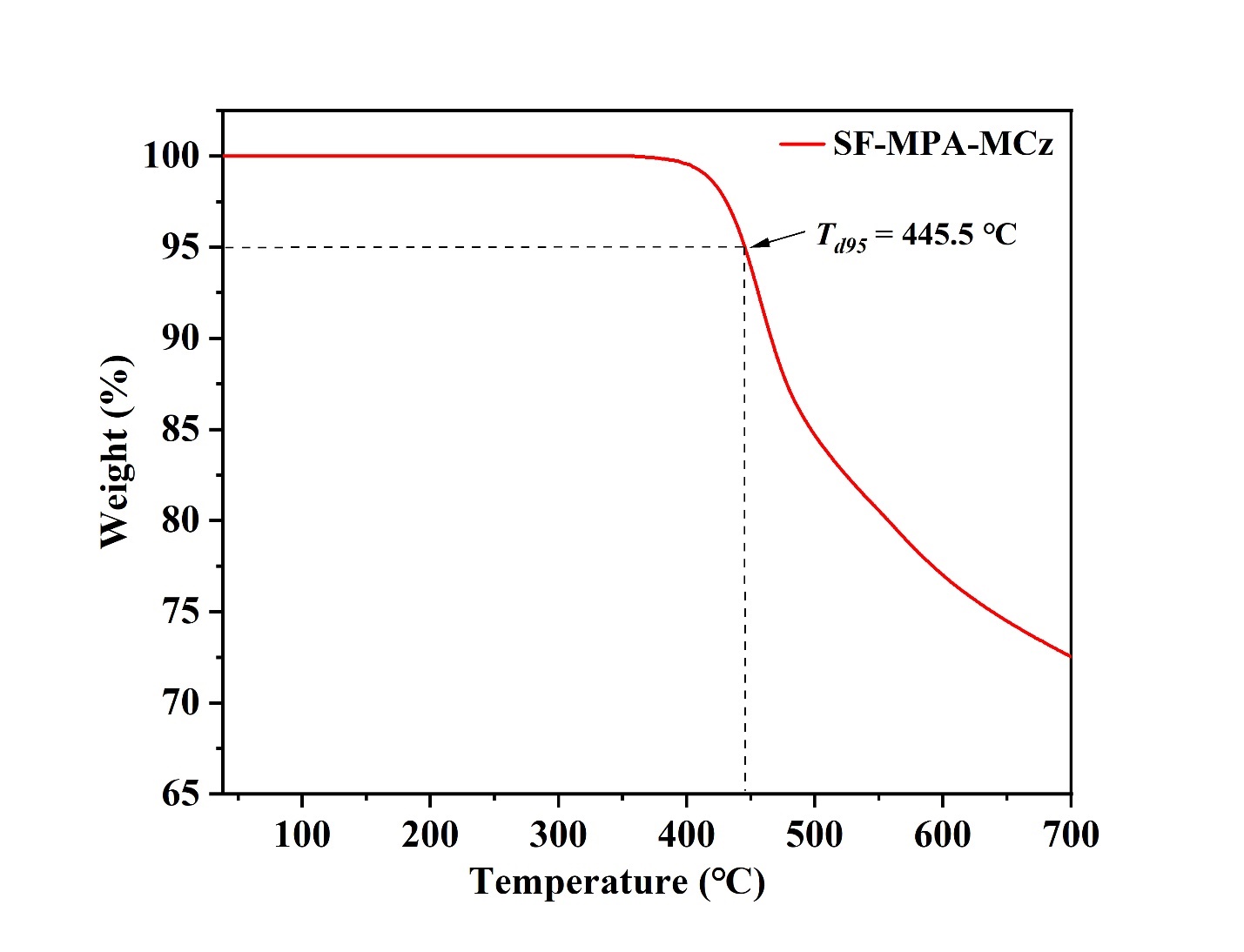


**Fig. S6.** Thermogravimetric measurement curve.


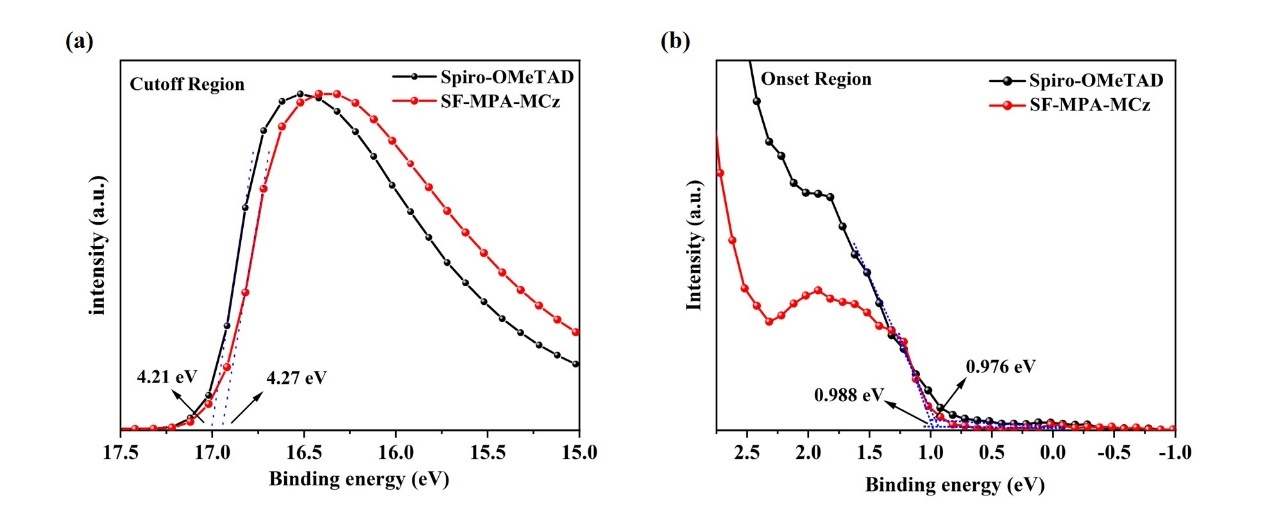


**Fig. S7.** UPS spectra of Spiro-OMeTAD and SF-MPA-MCz films.


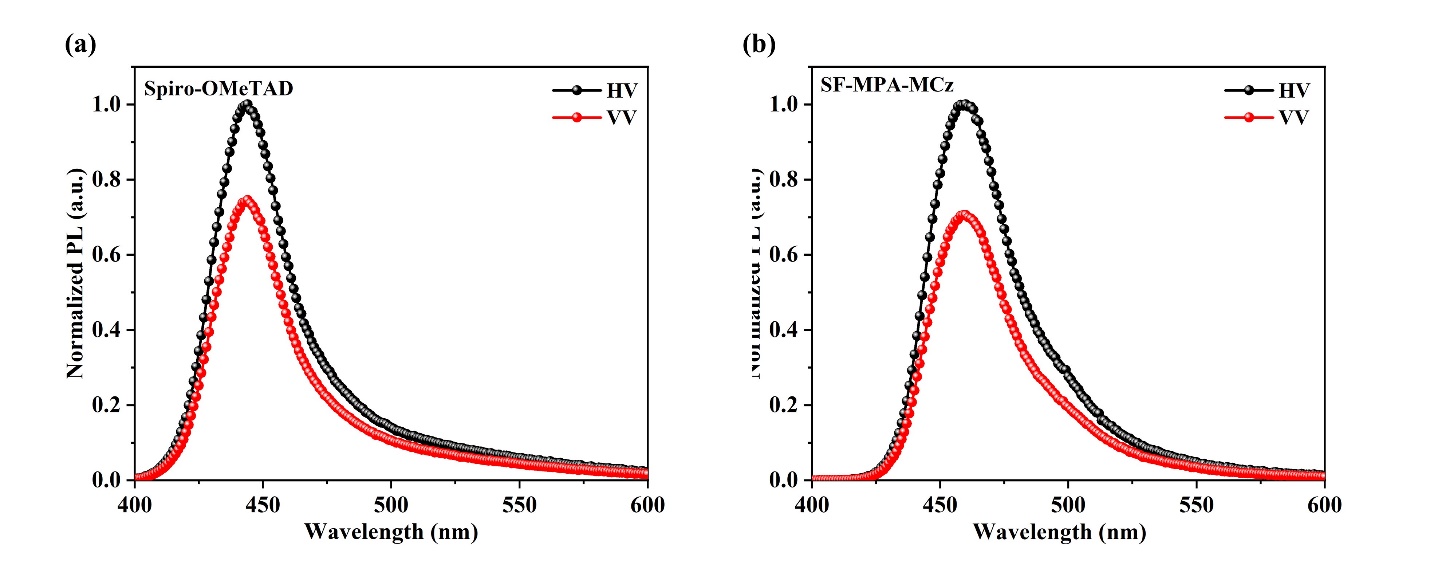


**Fig. S8.** Fluorescence anisotropy of (a) Spiro-OMeTAD and (b) SF-MPA-MCz films.


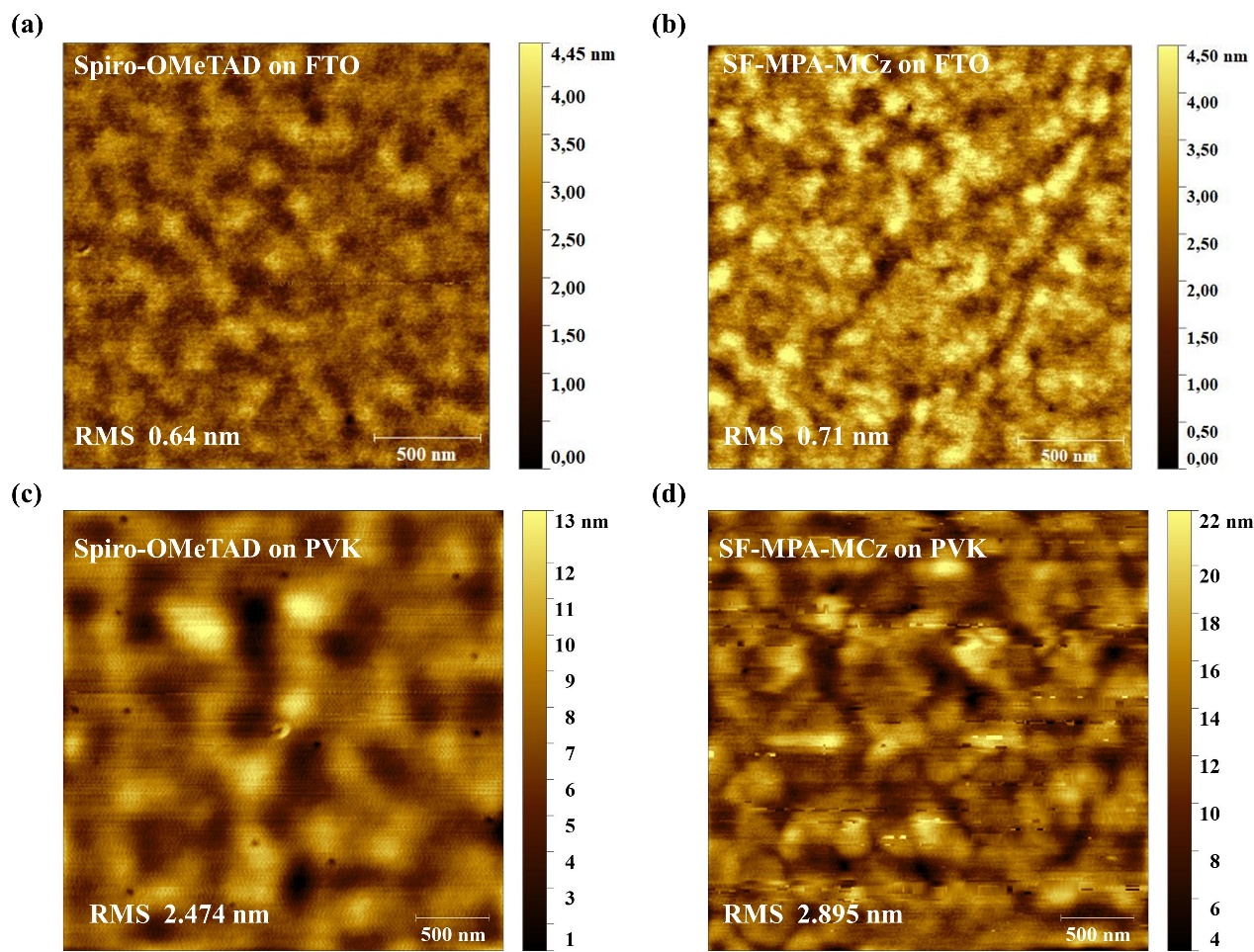


**Fig. S9.** AFM images of Spiro-OMeTAD and SF-MPA-MCZ films spin-coated on the FTO (a, b) and on the perovskite (c, d), respectively.


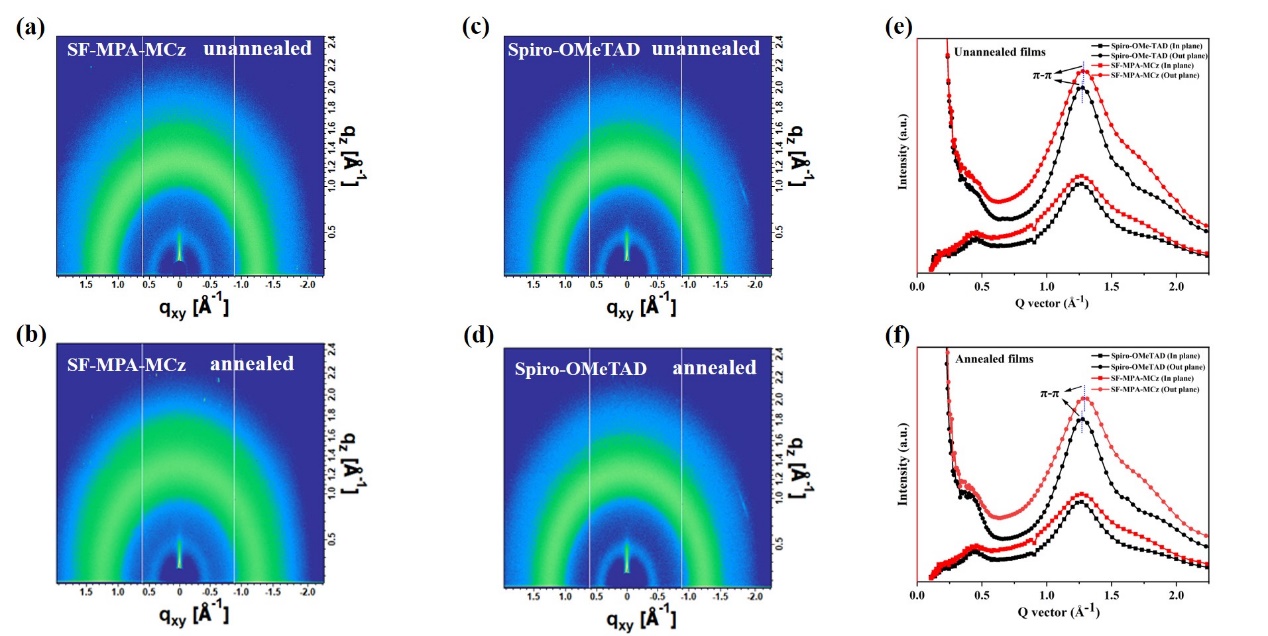


**Fig. S10.** 2D GIWAXS patterns of (**a**, **b**) SF-MPA-MCz, (**c**, **d**) Spiro-OMeTAD with or without annealing treatment, and (**e**, **f**) 1D GIWAXS profiles alone out-of-plane and in-plane direction.


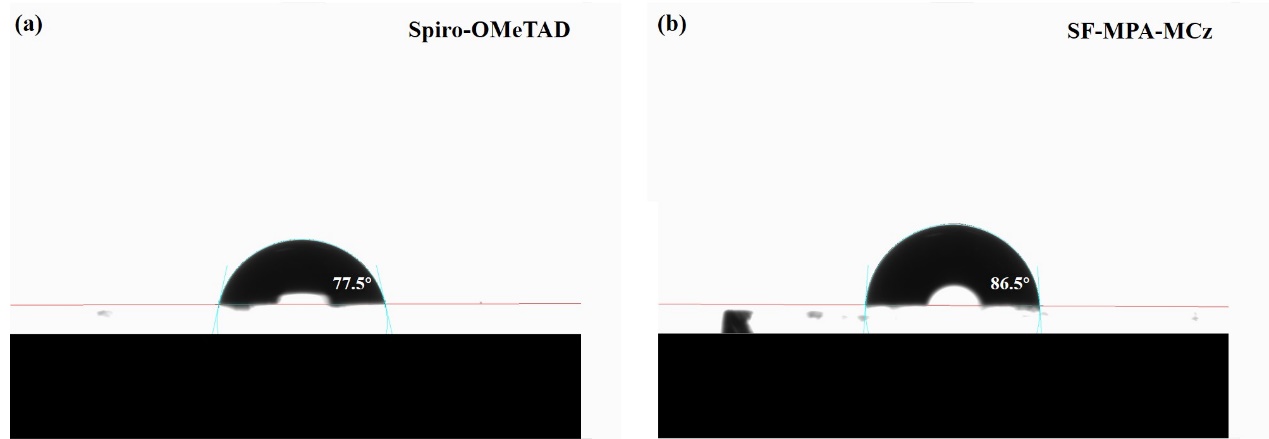


**Fig. S11.** Contact angles measurement of (**a**) Spiro-OMeTAD and (**b**) SF-MPA-MCz.


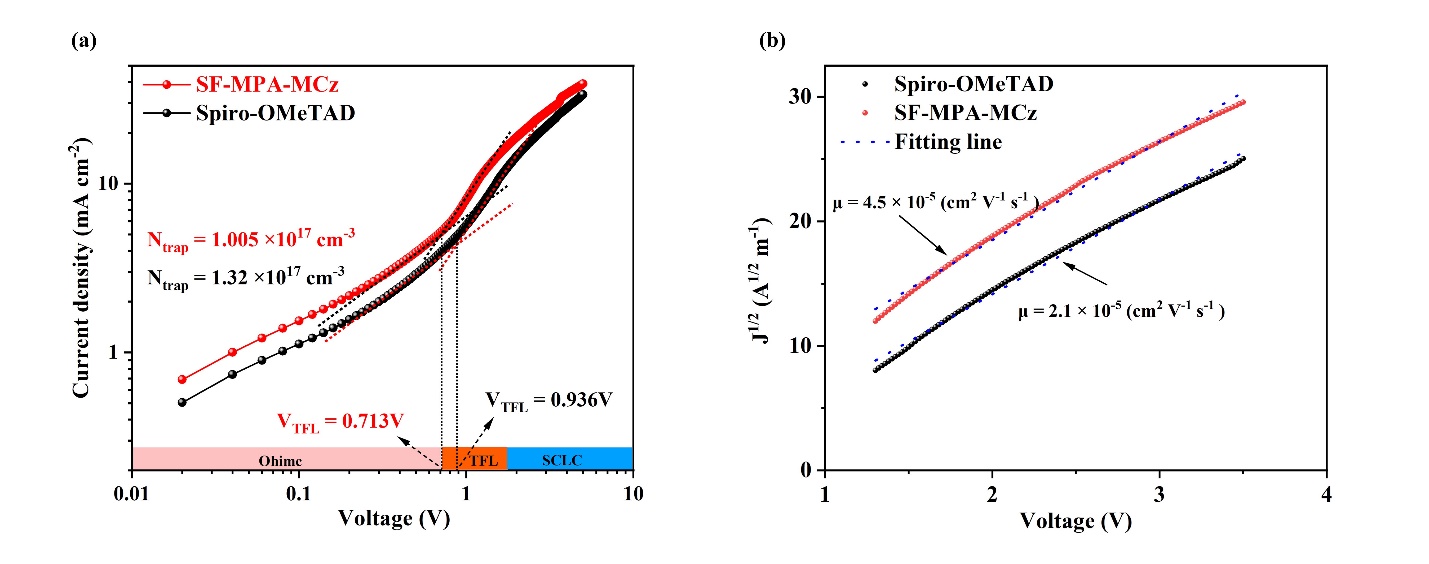


**Fig. S12.** *J-V* characteristics of trap-state densities (a) and hole mobility (b) obtained from hole-only devices measured by the space-charge-limited current (SCLC) method in the dark.


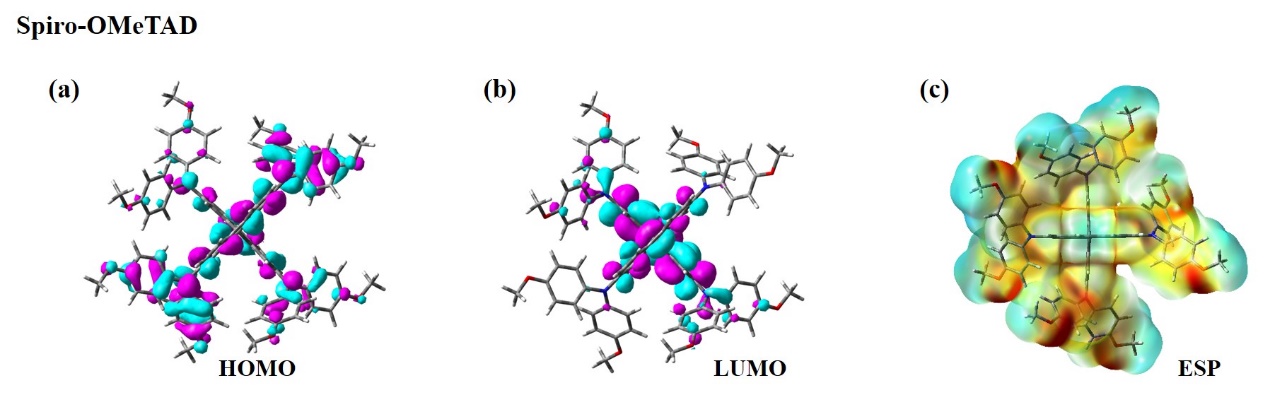


**Fig. S13.** Illustration of DFT calculated (**a**) highest occupied molecular orbital (HOMO), (**b**) lowest unoccupied molecular orbitals (LUMO), and (**c**) electrostatic surface potentials mapped for Spiro-OMeTAD.


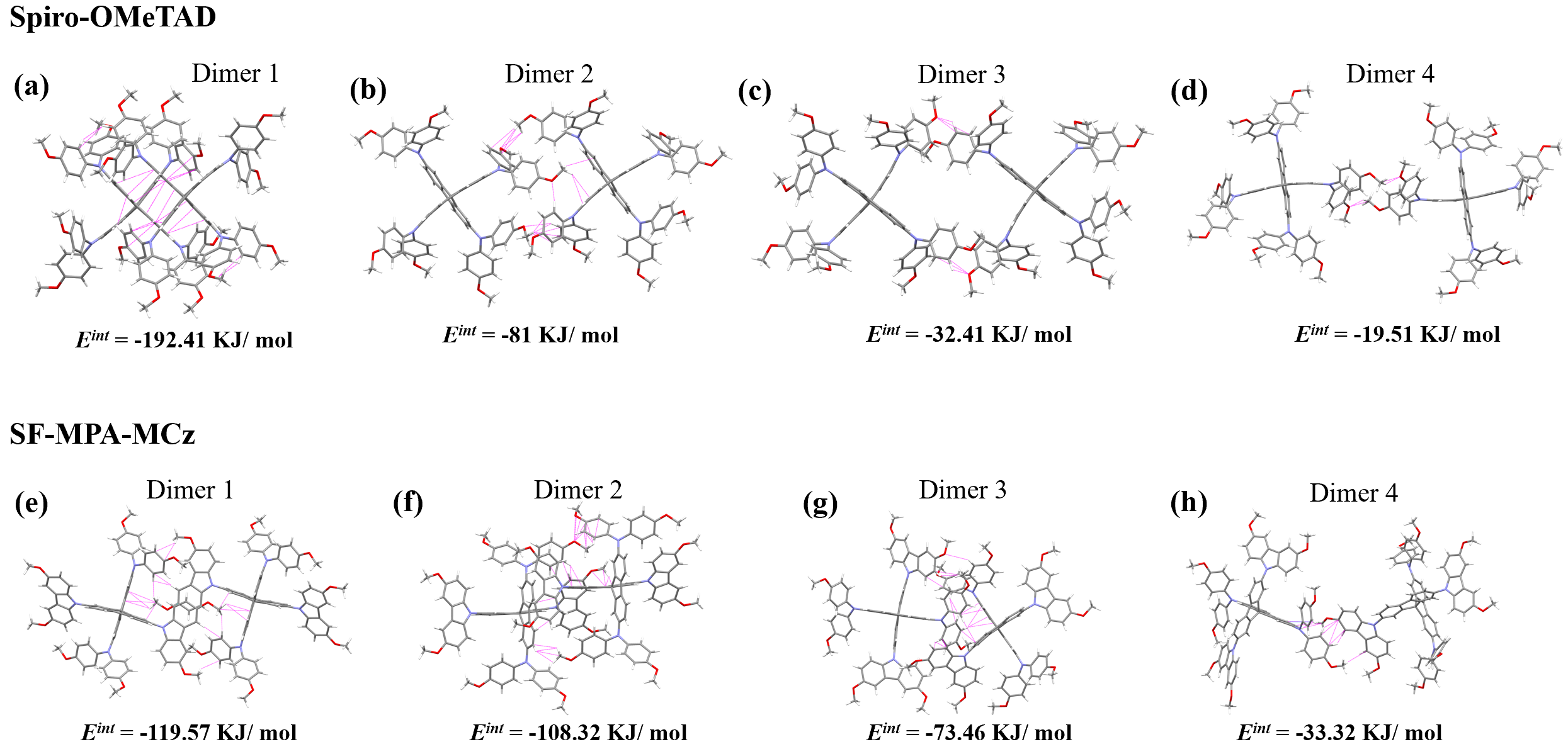


**Fig. S14.** Molecular packing structures and interaction energies of dimers 1−4 extracted from (**a**−**d**) Spiro-OMeTAD and (**e**−**h**) SF-MPA-MCz single crystals.


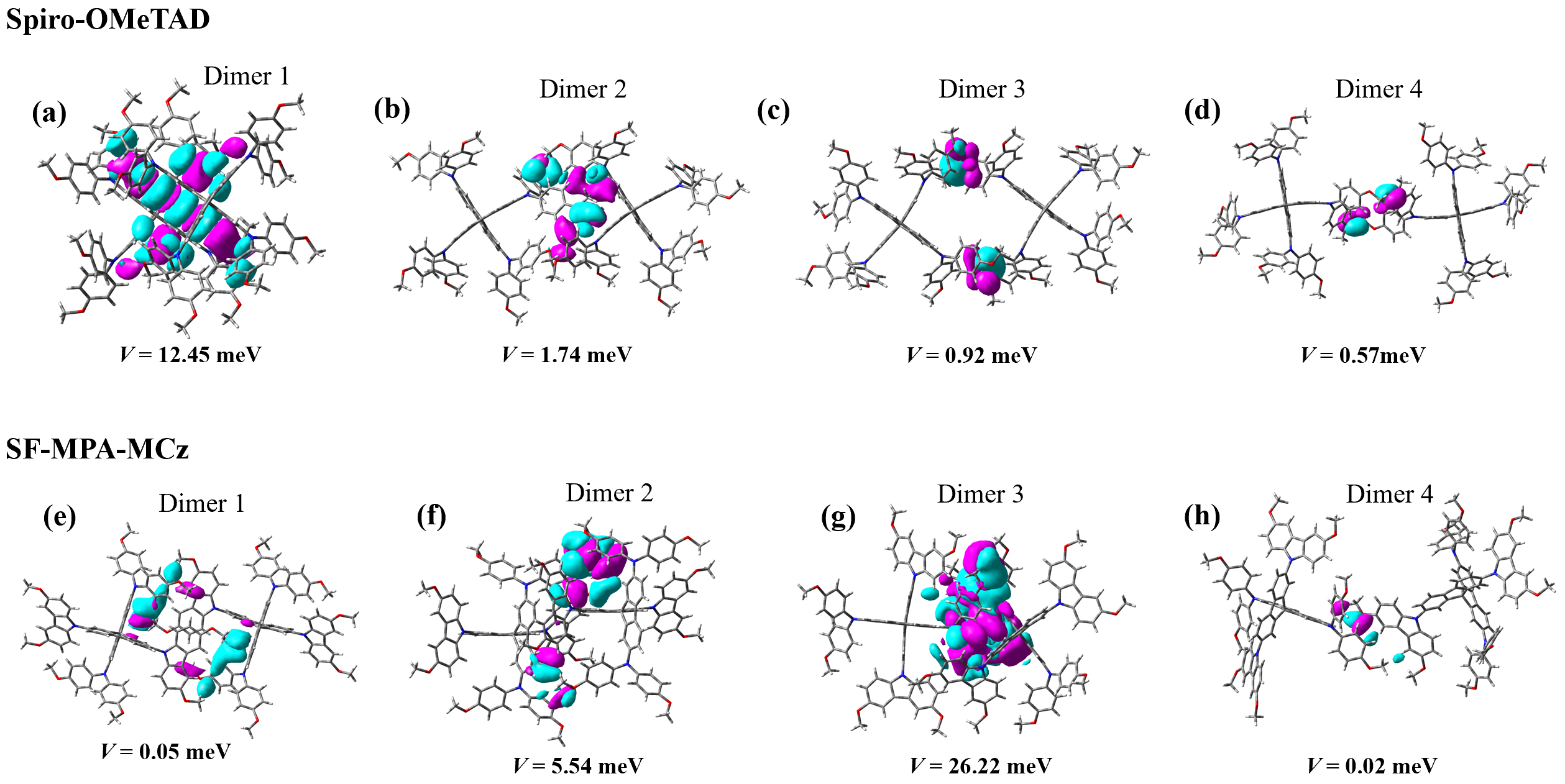


**Fig. S15.** Isodensity surface plots of intermolecular HOMO overlaps of dimers 1−4 extracted from (**a**−**d**) Spiro-OMeTAD and (**e**−**h**) SF-MPA-MCz single crystals.


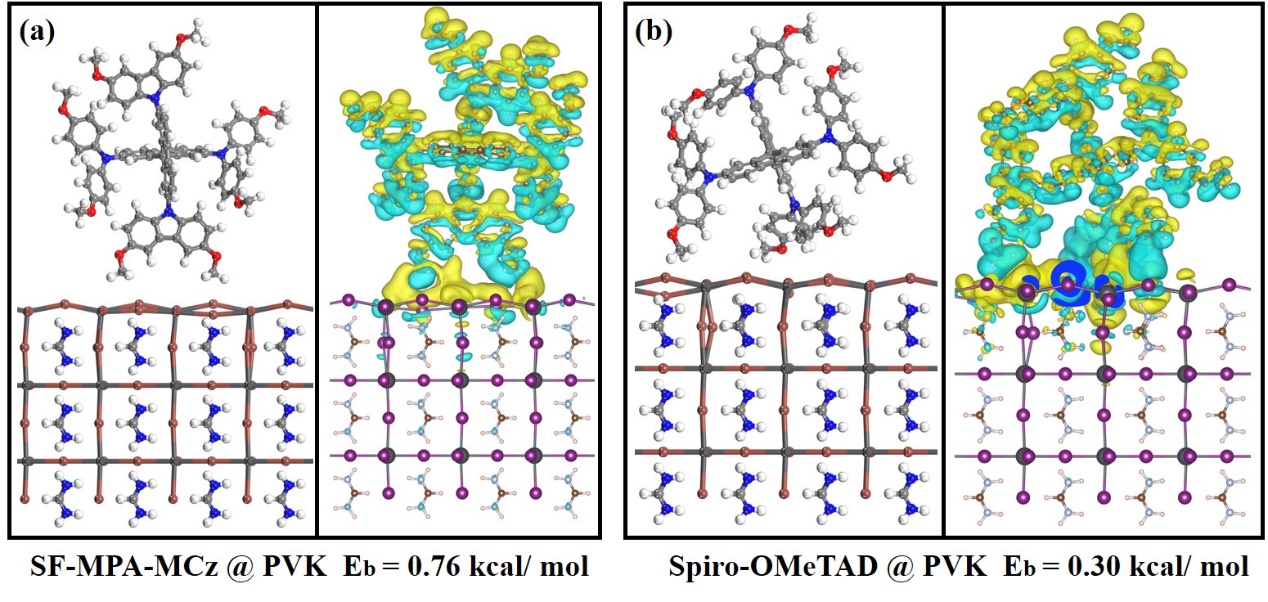


**Fig. S16.** DFT calculation of the interaction energies between (**a**) SF-MPA-MCz, (**b**) Spiro-OMeTAD, and the exposed perovskite surface.


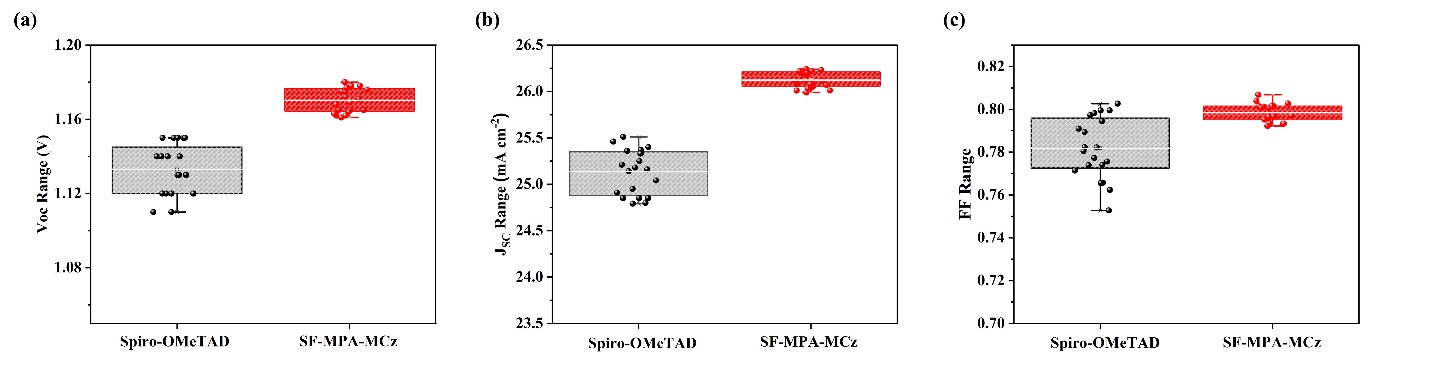


**Fig. S17.** (a) Voc, (b) Jsc, (c) FF distribution diagrams of devices (20 cells) with different HTMs.


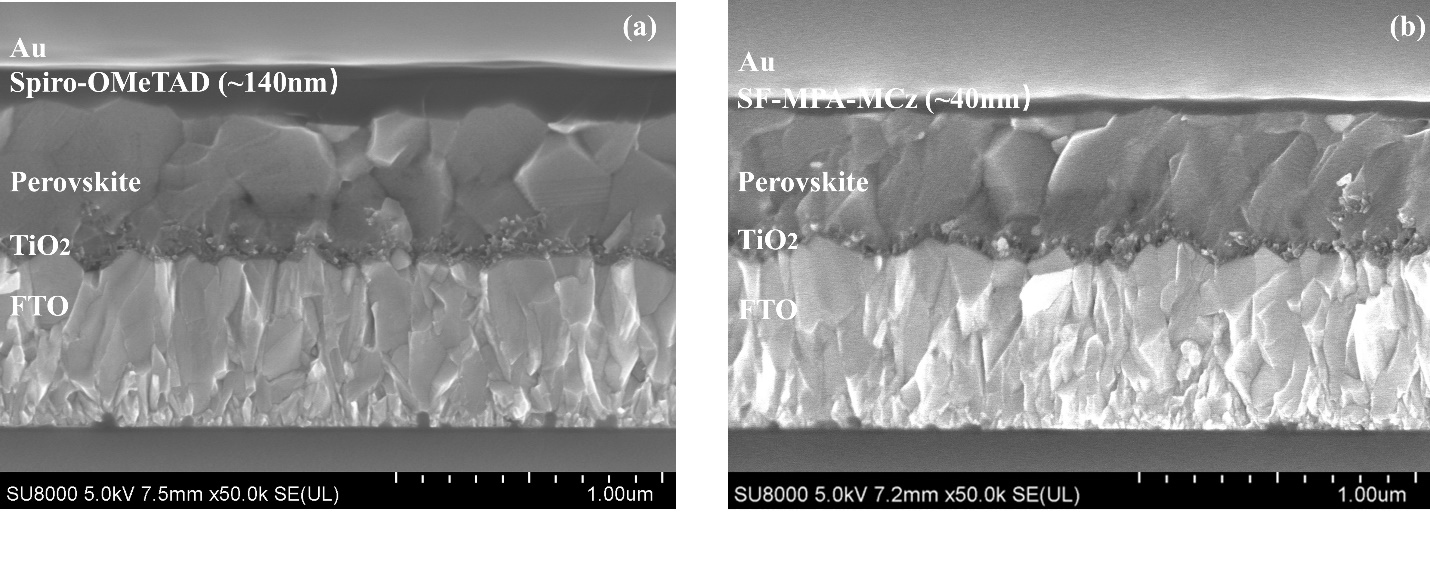


**Fig. S18.** (a) Cross-sectional SEM image of (**a**) Spiro-OMeTAD and (**b**) SF-MPA-MCz based device.


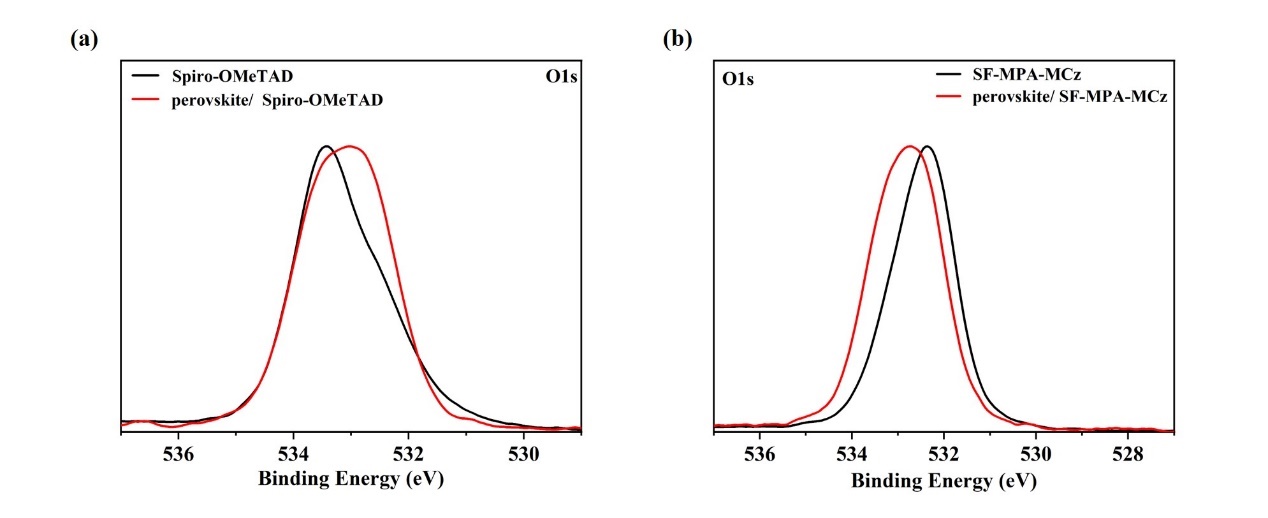


**Fig. S19.** O1s XPS spectra of perovskite films coated with (**a**) Spiro-OMeTAD and (**b**) SF-MPA-MCz.


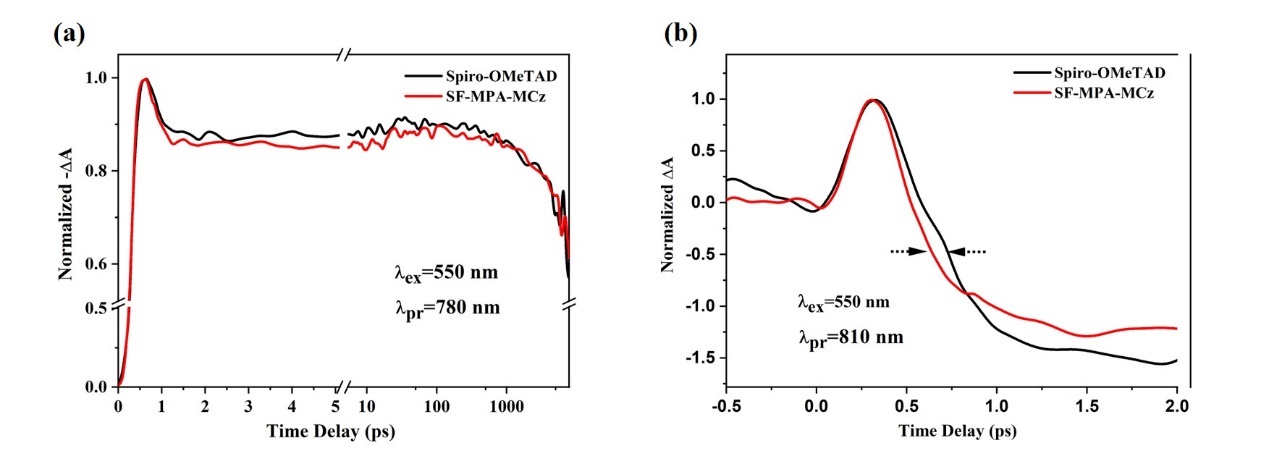


**Fig. S20.** (**a**) Carrier dynamics of perovskite films coated with Spiro-OMeTAD and SF-MPA-MCz excited by 550 nm. (**b**) Ultrafast carrier dynamics of photoinduced absorption at 810 nm of as-prepared samples.


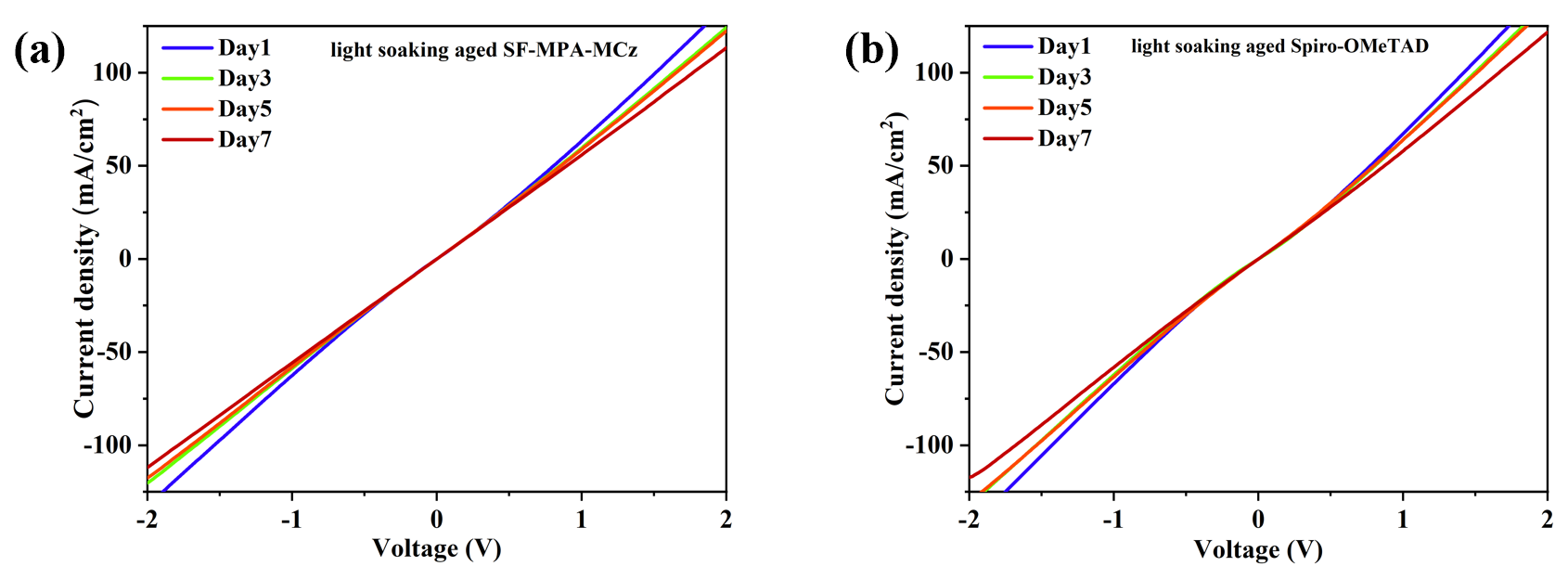


**Fig. S21.** The conductivity of (**a**) SF-MPA-MCz and (**b**) Spiro-OMeTAD films after light soaking aged.


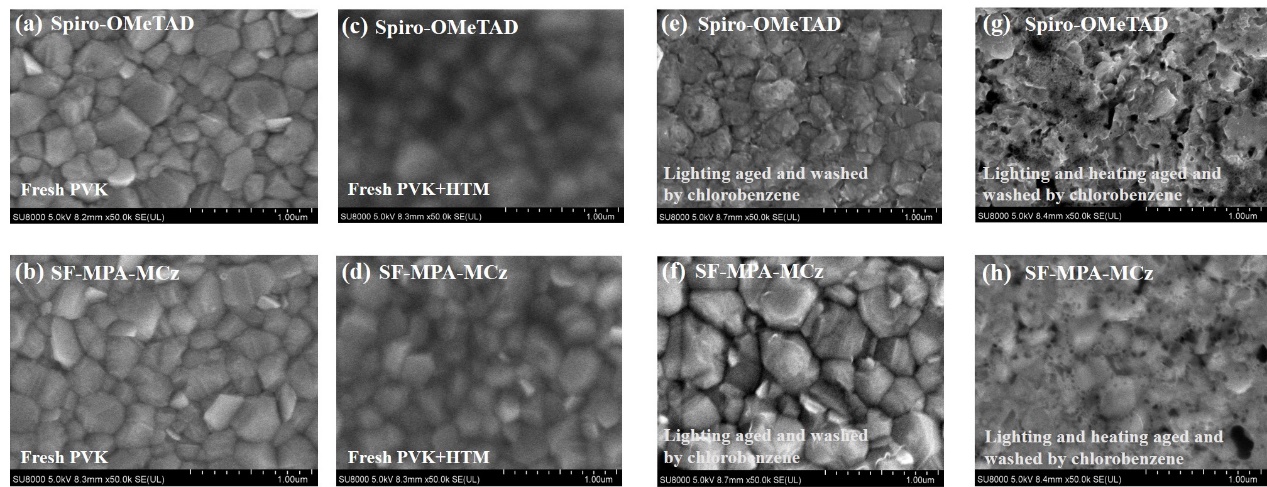


**Fig. S22.** Top-view SEM images of (**a**), (**b**) Fresh perovskite film, (**c**) Spiro-OMeTAD, and (**d**) SF-MPA-MCz coated perovskite. (**e**, **f**) light soaking aged perovskite washed by chlorobenzene. (**g**, **h**) light soaking and heating aged perovskite washed by chlorobenzene.

**Table S1.** Crystallographic parameters of SF-MPA-MCz.

| **Parameters** | **SF-MPA-MCz** |
| --- | --- |
| Empirical formula | C_81_H_64_N_4_O_8_ |
| Formula weight | 1220.47242 |
| Crystal color, habit | Yellow, flake |
| Crystal system | Triclinic |
| a, Å | 10.8155 |
| b, Å | 20.230 |
| c, Å | 28.680 |
| α, deg | 90.847 |
| β, deg | 94.666 |
| γ, deg | 91.096 |
| V, Å^3^ | 6252.2 |
| ρ calc, g/ cm^3^ | 1.298 |
| Space group | P -1 |
| Z value | 2 |
| Temperature, K | 200 |
| no. of reflections measured | 25515 |
| Residuals: R; wR2 | 0.1286/ 0.4212 |
| CCDC Nr. | 2297742 |

**Table S2.** The diffraction parameters of spiro-OMeTAD and SF-MPA-MCz.

|  | ***q_z_* (**Å **^-1^) unannealed film** | **Distance (**Å**)** | ***q_z_* (**Å **^-1^) annealed film** | **Distance (**Å**)** |
| --- | --- | --- | --- | --- |
| Spiro-OMeTAD | 1.272 | 4.9371 | 1.274 | 4.9293 |
| SF-MPA-MCz | 1.282 | 4.8985 | 1.295 | 4.8494 |

**Table S3.** DFT calculation data.

|  | **HOMO [eV]** | **LUMO [eV]** |
| --- | --- | --- |
| **SF-MPA-MCz** | -5.57 | -0.36 |
| **Spiro-OMeTAD** | -5.43 | 0.09 |

|  | ***E_H_*_−L_**  **[eV]** | ***E*_S1_**  **[eV]** | ***E*_b_**  **[eV]** | **Ip (*v*)**  **[eV]*^a^*** | **Ip (*a*)**  **[eV]*^b^*** | **HEP**  **[eV]** |
| --- | --- | --- | --- | --- | --- | --- |
| **SF-MPA-MCz** | 5.21 | 3.62 | 1.59 | 5.89 | 5.69 | 5.49 |
| **Spiro-OMeTAD** | 5.52 | 3.64 | 1.88 | 5.76 | 5.58 | 5.35 |

**Spiro-OMeTAD**

| **Dimers** | **E^ele^**  **[KJ/mol]** | **E^rep^**  **[KJ/mol]** | **E^disp^**  **[KJ/mol]** | **E^int^**  **[KJ/mol]** |
| --- | --- | --- | --- | --- |
| **Dimer 1** | 11.63 | 107.68 | -311.71 | -192.41 |
| **Dimer 2** | -14.93 | 42.20 | -108.27 | -81.00 |
| **Dimer 3** | 5.93 | 31.87 | -60.20 | -32.41 |
| **Dimer 4** | -4.19 | 17.39 | -32.71 | -19.51 |

**SF-MPA-MCz**

| **Dimers** | ***V***  **[meV]** | ***d***  **[Å]** | ***k***  **[s^-1^]** | ***λ***  **[eV]** | ***µ*_h_**  **[cm^2^ V^-1^s^-1^]** |
| --- | --- | --- | --- | --- | --- |
| **Dimer 1** | 0.05 | 16.51 | 1.34×10^6^ | 0.40 | 0.025 |
| **Dimer 2** | 5.54 | 10.81 | 1.65×10^10^ |  |  |
| **Dimer 3** | 26.22 | 10.46 | 3.70×10^13^ |  |  |
| **Dimer 4** | 0.02 | 19.57 | 2.15×10^5^ |  |  |

**Spiro-OMeTAD**

| **Dimers** | ***V***  **[meV]** | ***d***  **[Å]** | ***k***  **[s^-1^]** | ***λ***  **[eV]** | ***µ*_h_**  **[cm^2^ V^-1^s^-1^]** |
| --- | --- | --- | --- | --- | --- |
| **Dimer 1** | 12.45 | 9.52 | 7.47×10^10^ | 0.41 | 0.0043 |
| **Dimer 2** | 1.74 | 14.72 | 1.46×10^9^ |  |  |
| **Dimer 3** | 0.92 | 14.27 | 4.08×10^8^ |  |  |
| **Dimer 4** | 0.57 | 17.42 | 1.57×10^8^ |  |  |

**Table S4.** Comparison of material dosage, Li-TFSI doping concentration, and device efficiency for Spiro-type molecules.

|  | **Structure** | **Hole Mobility**  **(cm^2^ V^−1^ s ^−1^)** | **HTMs Concentration**  **(mg mL^−1^)** | **Li-TFSI**  **dopant Concentration**  **(mg mL^−1^)** | **PCE**  **(%)** | **Ref** |
| --- | --- | --- | --- | --- | --- | --- |
| **Spiro-Naph** | 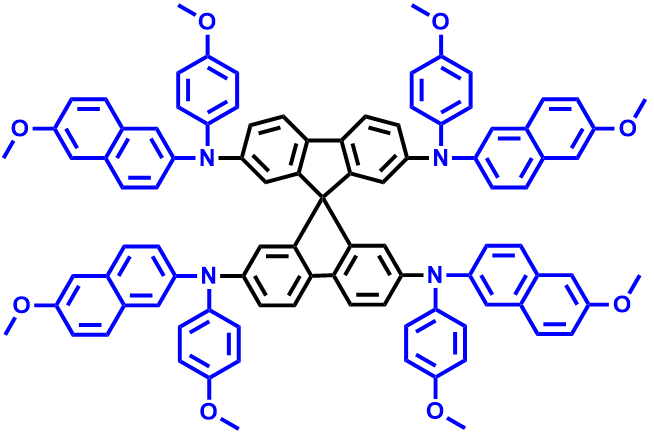 | **8.08 × 10^−3^** | **90.9** | **520 acetonitrile/**  **(18 μL)** | **24.43** | (*11*) |
| **DM** | 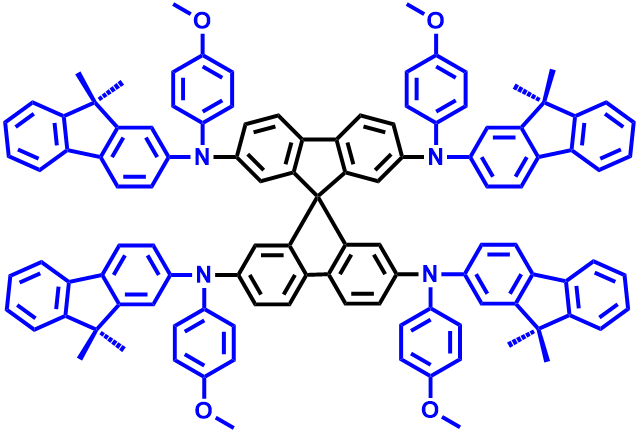 | **~10^-3^** | **28.6** | **340 CB/ (10 μL)** | **22.2** | (*12*) |
| **SC** | 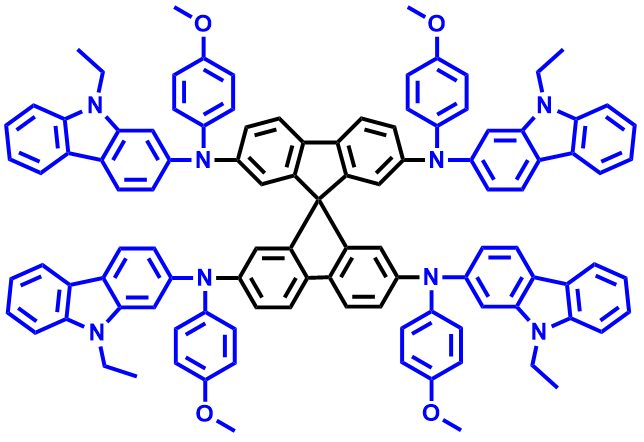 | **3.15 × 10^−3^** | **46.3** | **170 CB/ (54 μL)** | **21.76** | (*13*) |
| **ST** | 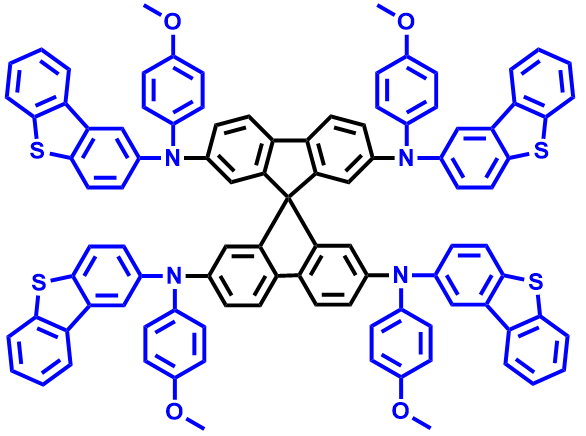 | **1.77 × 10^−3^** | **46.3** | **170 CB/ (54 μL)** | **18.18** |  |
| **spiro-DBF** | 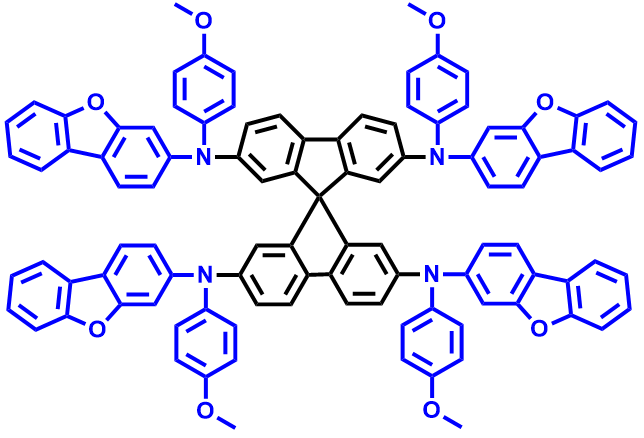 | **6.31 × 10^-3^** | **70** | **520 acetonitrile/**  **(9 μL)** | **21.43** | (*14*) |
| **spiro-DBT** | 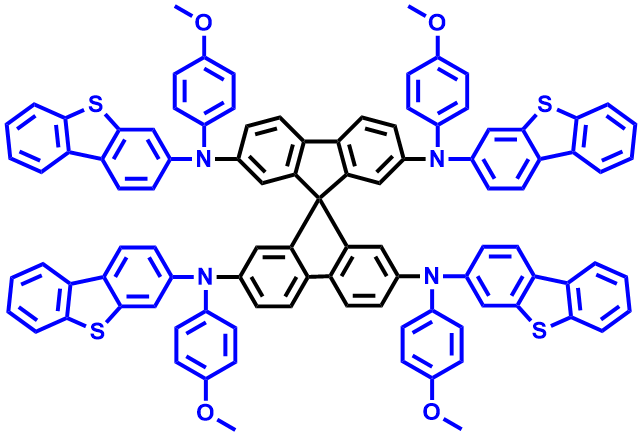 | **1.25 × 10^-3^** | **70** | **520 acetonitrile/**  **(9 μL)** | **20.37** |  |
| **Spiro-mF** | 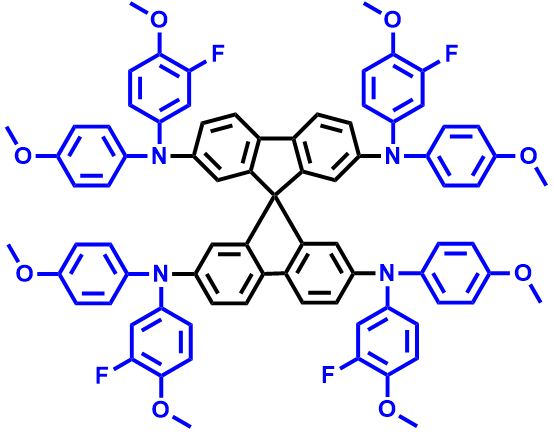 | **7.47 × 10^–3^** | **90.9** | **516 acetonitrile/**  **(29 μL)** | **24.82** | (*15*) |
| **Spiro-oF** | 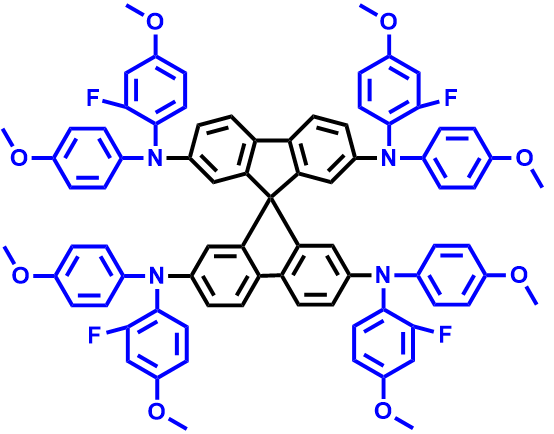 | **7.29 × 10^–3^** | **90.9** | **516 acetonitrile/**  **(17 μL)** | **22.78** |  |
| **V1307** | 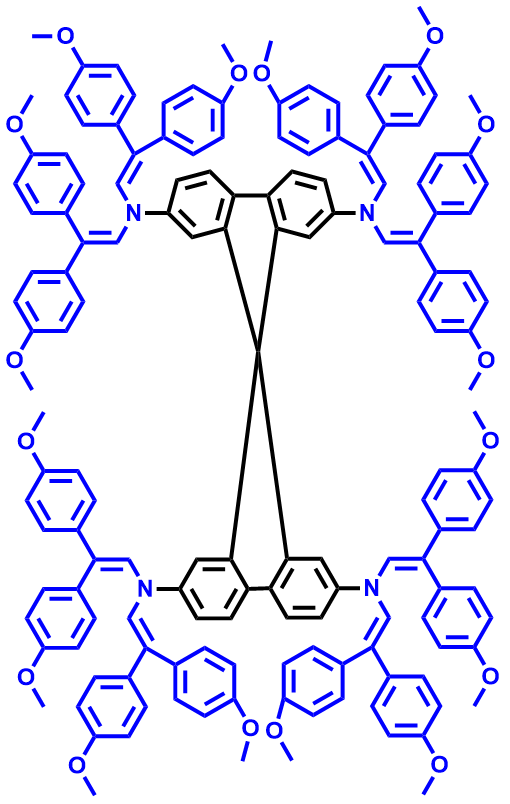 | **6.4 × 10^-4^** | **20 mM (47mg/ mL)** | **1.8M acetonitrile/**  **(0.5 molar ratio for HTMs)** | **19.2** | (*16*) |
| **V1267** | 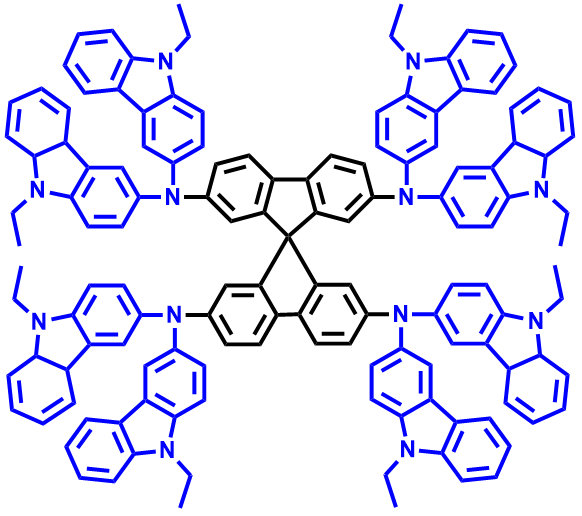 | **3.4 × 10^-4^** | **20 mM (38.6 mg/ mL)** | **1.8M acetonitrile**  **(0.5 molar ratio for HTMs)** | **18.3** | (*17*) |
| **V1240** | 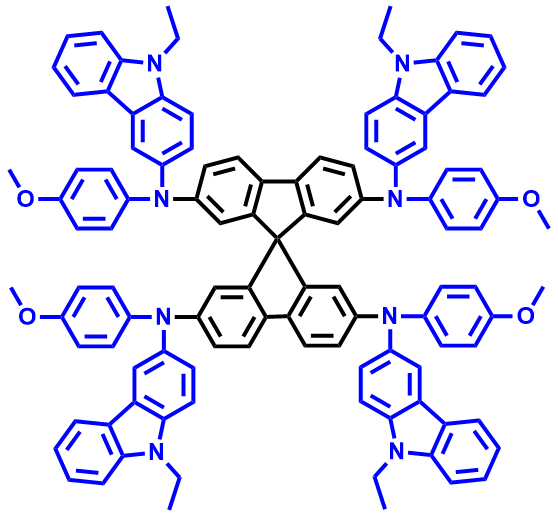 | **5.6 × 10^-6^** | **20 mM (31.5 mg/ mL)** | **1.8M acetonitrile**  **(0.5 molar ratio for HTMs)** | **17.6** |  |
| **Spiro-4TFETAD** | 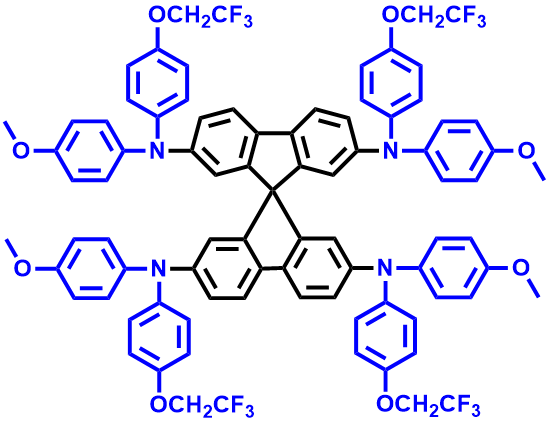 | **2.04 × 10^-4^** | **90** | **520 acetonitrile/**  **(22 μL)** | **21.11** | (*18*) |
| **1** | 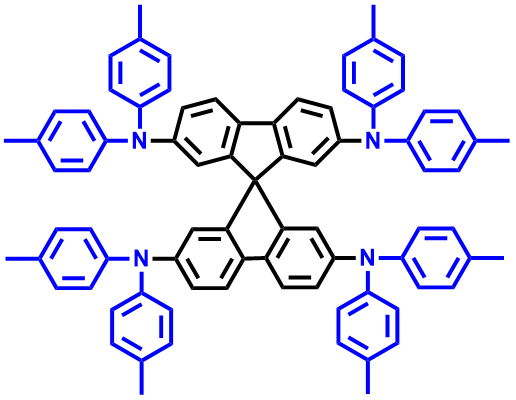 | **5 × 10^-3^** | **37 mM (44.2mg/ mL)** | **6.6 molar ratio for HTMs** | **21.87** | (*19*) |
| **2** | 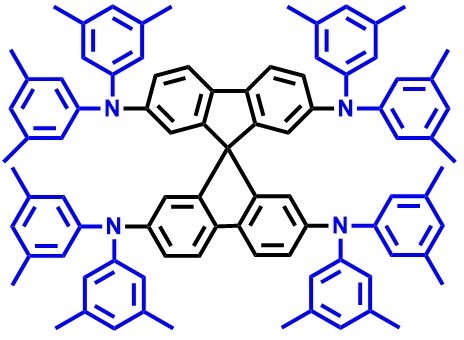 | **5 × 10^-3^** | **37 mM (40.6mg/ mL)** | **6.6 molar ratio for HTMs** | **21.66** |  |
| **Spiro**  **-BD-2OEG** | 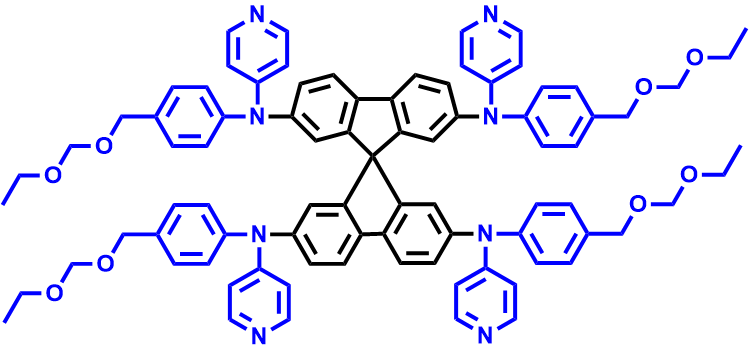 | **3.87 ×10^-4^** | **1mg/ mL as an additive** | **260 acetonitrile**  **(35 μL)** | **24.19** | (*20*) |
| **SF-MPA-MCz** | 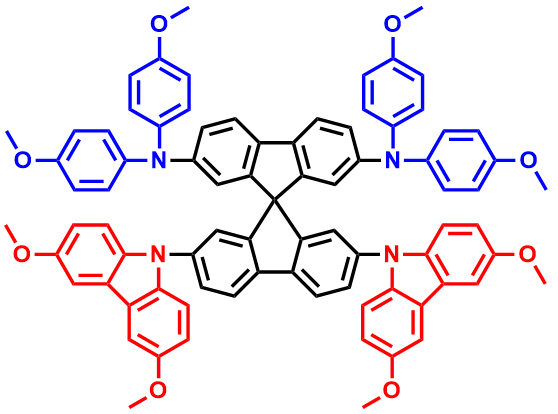 | **4.5 ×10^-5^** | **30** | **520 acetonitrile/**  **(10 μL)** | **24.53** | **This work** |

**Table S5.** the parameter of electrochemical impedance spectroscopy.

|  | R_S_ (Ω) | R_CT_ (Ω) | R_rec_ (Ω) |
| --- | --- | --- | --- |
| Spiro-OMeTAD | 5.22 | 95 | 1258 |
| SF-MPA-MCz | 4.16 | 73 | 1962 |

**References**

1. S. H. Lee, T. Nakamura, T. Tsutsui, Synthesis and Characterization of Oligo(9,9-dihexyl-2,7-fluorene ethynylene)s: For Application as Blue Light-Emitting Diode. *Org. Lett.* **3**, 2005–2007 (2001).

2. T. Lu, F. Chen, Multiwfn: A multifunctional wavefunction analyzer. *J. Comput. Chem.* **33**, 580–592 (2012).

3. Q. Wang, E. Mosconi, C. Wolff, J. Li, D. Neher, F. De Angelis, G. P. Suranna, R. Grisorio, A. Abate, Rationalizing the Molecular Design of Hole‐Selective Contacts to Improve Charge Extraction in Perovskite Solar Cells. *Advanced Energy Materials*. **9**, 1900990 (2019).

4. J. P. Perdew, K. Burke, M. Ernzerhof, Generalized Gradient Approximation Made Simple. *Phys. Rev. Lett.* **77**, 3865–3868 (1996).

5. R. A. Vargas−Hernández, Bayesian Optimization for Calibrating and Selecting Hybrid-Density Functional Models. *J. Phys. Chem. A*. **124**, 4053–4061 (2020).

6. Hongzhiwei Technology, Device Studio, Version 2023A, China, 2023. Available online:https://iresearch.net.cn/cloudSoftware

7. W. Liu, G. Hong, D. Dai, L. Li, M. Dolg, The Beijing four-component density functional program package (BDF) and its application to EuO, EuS, YbO and YbS. *Theoretical Chemistry Accounts: Theory, Computation, and Modeling (Theoretica Chimica Acta)*. **96**, 75–83 (1997).

8. Y. Zhang, B. Suo, Z. Wang, N. Zhang, Z. Li, Y. Lei, W. Zou, J. Gao, D. Peng, Z. Pu, Y. Xiao, Q. Sun, F. Wang, Y. Ma, X. Wang, Y. Guo, W. Liu, BDF: A relativistic electronic structure program package. *The Journal of Chemical Physics*. **152**, 064113 (2020).

9. P. E. Blöchl, Projector augmented-wave method. *Phys. Rev. B*. **50**, 17953–17979 (1994).

10. K. Momma, F. Izumi, *VESTA 3* for three-dimensional visualization of crystal, volumetric and morphology data. *J Appl Crystallogr*. **44**, 1272–1276 (2011).

11. M. Jeong, I. W. Choi, K. Yim, S. Jeong, M. Kim, S. J. Choi, Y. Cho, J.-H. An, H.-B. Kim, Y. Jo, S.-H. Kang, J.-H. Bae, C.-W. Lee, D. S. Kim, C. Yang, Large-area perovskite solar cells employing spiro-Naph hole transport material. *Nat. Photon.* **16**, 119–125 (2022).

12. N. J. Jeon, H. Na, E. H. Jung, T.-Y. Yang, Y. G. Lee, G. Kim, H.-W. Shin, S. Il Seok, J. Lee, J. Seo, A fluorene-terminated hole-transporting material for highly efficient and stable perovskite solar cells. *Nat Energy*. **3**, 682–689 (2018).

13. Z. Deng, M. He, Y. Zhang, F. Ullah, K. Ding, J. Liang, Z. Zhang, H. Xu, Y. Qiu, Z. Xie, T. Shan, Z. Chen, H. Zhong, C.-C. Chen, Design of Low Crystallinity Spiro-Typed Hole Transporting Material for Planar Perovskite Solar Cells to Achieve 21.76% Efficiency. *Chem. Mater.* **33**, 285–297 (2021).

14. Y. Liang, J. Chen, X. Zhang, M. Han, R. Ghadari, N. Wu, Y. Wang, Y. Zhou, X. Liu, S. Dai, Dibenzo heterocyclic-terminated spiro-type hole transporting materials for perovskite solar cells. *J. Mater. Chem. C*. **10**, 10988–10994 (2022).

15. M. Jeong, I. W. Choi, E. M. Go, Y. Cho, M. Kim, B. Lee, S. Jeong, Y. Jo, H. W. Choi, J. Lee, J.-H. Bae, S. K. Kwak, D. S. Kim, C. Yang, Stable perovskite solar cells with efficiency exceeding 24.8% and 0.3-V voltage loss. *Science*. **369**, 1615–1620 (2020).

16. D. Vaitukaityte, C. Momblona, K. Rakstys, A. A. Sutanto, B. Ding, C. Igci, V. Jankauskas, A. Gruodis, T. Malinauskas, A. M. Asiri, P. J. Dyson, V. Getautis, M. K. Nazeeruddin, Cut from the Same Cloth: Enamine-Derived Spirobifluorenes as Hole Transporters for Perovskite Solar Cells. *Chem. Mater.* **33**, 6059–6067 (2021).

17. A. Jegorovė, C. Momblona, M. Daškevičienė, A. Magomedov, R. Degutyte, A. M. Asiri, V. Jankauskas, A. A. Sutanto, H. Kanda, K. Brooks, N. Klipfel, M. K. Nazeeruddin, V. Getautis, Molecular Engineering of Fluorene‐Based Hole‐Transporting Materials for Efficient Perovskite Solar Cells. *Solar RRL*. **6**, 2100990 (2022).

18. Z. Zhang, L. Yuan, B. Li, H. Luo, S. Wang, Z. Li, Y. Xing, J. Wang, P. Dong, K. Guo, Z. Wang, K. Yan, A Trifluoroethoxyl Functionalized Spiro‐Based Hole‐Transporting Material for Highly Efficient and Stable Perovskite Solar Cells. *Solar RRL*. **6**, 2100944 (2022).

19. X. Sallenave, M. Shasti, E. H. Anaraki, D. Volyniuk, J. V. Grazulevicius, S. M. Zakeeruddin, A. Mortezaali, M. Grätzel, A. Hagfeldt, G. Sini, Interfacial and bulk properties of hole transporting materials in perovskite solar cells: spiro-MeTAD *versus* spiro-OMeTAD. *J. Mater. Chem. A*. **8**, 8527–8539 (2020).

20. H. Yang, Y. Shen, R. Zhang, Y. Wu, W. Chen, F. Yang, Q. Cheng, H. Chen, X. Ou, H. Yang, F. Gao, Y. Li, Y. Li, Composition‐Conditioning Agent for Doped Spiro‐OMeTAD to Realize Highly Efficient and Stable Perovskite Solar Cells. *Advanced Energy Materials*. **12**, 2202207 (2022).
